# Multichromophoric photoswitches for solar energy storage: from azobenzene to norbornadiene, and MOST things in between

**DOI:** 10.1039/d3ta05972c

**Published:** 2024-01-11

**Authors:** Rebecca J. Salthouse, Kasper Moth-Poulsen

**Affiliations:** a Department of Chemical Engineering, Universitat Politècnica de Catalunya, EEBE Eduard Maristany 16 08019 Barcelona Spain kasper.moth-poulsen@upc.edu; b Catalan Institution for Research & Advanced Studies, ICREA Pg. Llu'ıs Companys 23 Barcelona Spain; c Institute of Materials Science of Barcelona, ICMAB-CSIC Bellaterra Barcelona 08193 Spain; d Department of Chemistry and Chemical Engineering, Chalmers University of Technology Kemivagen 4 Gothenburg 412 96 Sweden

## Abstract

The ever-increasing global demands for energy supply and storage have led to numerous research efforts into finding and developing renewable energy technologies. Molecular solar thermal energy storage (MOST) systems utilise molecular photoswitches that can be isomerized to a metastable high-energy state upon solar irradiation. These high-energy isomers can then be thermally or catalytically converted back to their original state, releasing the stored energy as heat on-demand, offering a means of emission-free energy storage from a closed system, often from only organic materials. In this context, multichromophoric systems which incorporate two or more photochromic units may offer additional functionality over monosubstituted analogues, due to their potential to access multiple states as well as having more attractive physical properties. The extended conjugation offered by these systems can lead to a red shift in the absorption profile and hence a better overlap with the solar spectrum. Additionally, the multichromophoric design may lead to increased energy storage densities due to some of the molecular weight being ‘shared’ across several energy storage units. This review provides an overview and analysis of multichromophoric photoswitches incorporating the norbornadiene/quadricyclane (NBD/QC) couple, azobenzene (AZB), dihydroazulene (DHA) and diarylethene (DAE) systems, in the context of energy storage applications. Mixed systems, where two or more different chromophores are linked together in one molecule, are also discussed, as well as limitations such as the loss of photochromism due to inner filter effects or self-quenching, and how these challenges may be overcome in future designs of multichromophoric systems.

## Abstract 2

In the context of energy storage, multichromophoric systems may offer additional functionality over monosubstituted analogues due to their potential to access multiple states as well as having more attractive physical properties.

## Graphical abstract

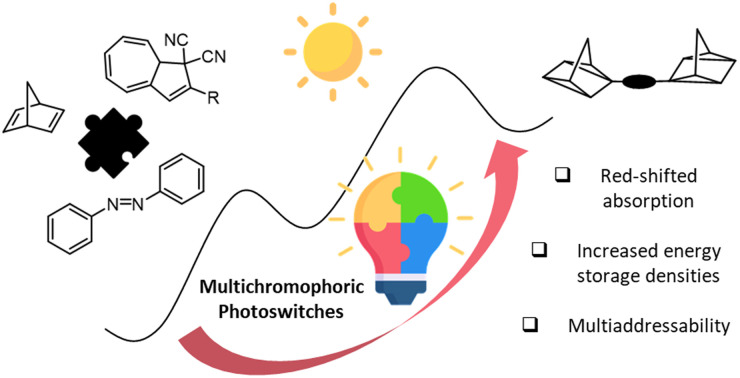

## Introduction

Molecular solar thermal (MOST) systems, also known as solar thermal fuels (STFs), comprised of a photoswitchable molecule with a higher energy metastable photoisomer, represent a promising avenue for harvesting and storing solar energy in a renewable fashion, whilst offering a means of emission-free energy storage from a closed system.^1,2^ The first examples of MOST systems date back to the early 1900s, where Luther and Weigert identified anthracene dimerization as a possible means of solar energy storage.^3^ They commented that this was a completely abiotic process and that an estimated 5% of the solar energy could be recovered as heat.^4^ Since then, several different molecular systems have been explored, including norbornadienes (NBD),^5^ azobenzenes (AZB)^6–8^ and dihydroazulene–vinylheptafulvene (DHA–VHF) systems.^9^ Many advancements have been made in the MOST field over the last decade with regards to molecular design,^10,11^ as well as the implementation and engineering of devices.^1,12^

However, in order for these MOST systems to become a practically viable renewable energy solution, certain key properties need to be engineered such as the solar spectrum match and energy density to harvest and store as much of the sun's energy as possible. Multichromophoric photoswitches, where there are two or more photochromic units within the same molecule, offer several possible advantages in this regard; the extended π-conjugation of these systems leads to more red-shifted absorption profiles and hence better overlap with the solar spectrum. The multi-switch nature also means that part of the molecular weight is ‘shared’ between several energy storage units, leading to high energy storage densities amongst reported MOST systems.^13^ Moreover, some dendrimer-like multiphotochromic systems have been shown to possess increased energy densities and storage times due to various ‘supramolecular’ effects.^14^

Multichromophoric systems involving two different types of photoswitches^15^ have also been explored. These mixed-multiphotochromic systems come with their own challenges, such as inhibition of switching due to inner filter effects or intramolecular energy transfer if the two scaffolds are energetically coupled. If these challenges are addressed, multiphotochromic systems may lead to a better coverage of the solar spectrum and higher energy densities, as well as new or better opportunities for ‘smart’ materials due their multiaddressable properties. These factors together motivate this review in which we present and discuss the growing literature on multiphotochromic systems for solar energy storage.^16^

**Scheme 1:**
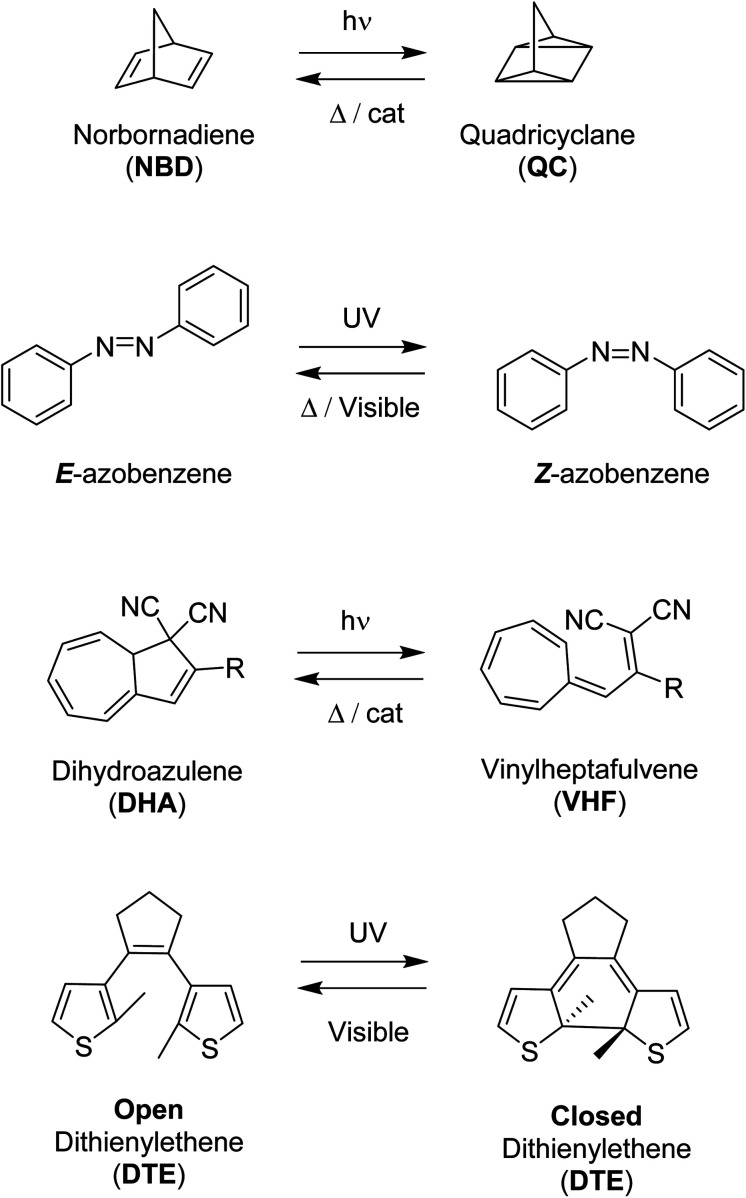
Examples of photoswitches and their respective photoisomers.

A selection of photoswitches and their photoisomers that will form the parts of the multiphotochromic systems discussed in this review, are outlined in Scheme 1. Azobenzene undergoes isomerization from the stable *E* (*trans*) isomer to the higher energy *Z* (*cis*) isomer upon irradiation with UV light. The photoisomerization of dihydroazulene (DHA) to its photoisomer vinylheptafulvene (VHF) occurs *via* a carbon–carbon bond-breaking ring opening event. Diarylethenes such as diethienylethene (DTE) can be switched by inducing an electrocyclisation process from its colourless open form featuring a hexatriene core, to the coloured closed form due to the extended π-system formed upon ring closure. Both DHA and diarylethenes are positive photochromic systems, *i.e.* a red-shift in the absorption profile occurs upon photoisomerization, as well as azobenzene which usually forms an additional red-shifted n–π* band for the higher-energy *Z* photoisomer. On the contrary, the mode of action of the negative photochromic norbornadiene (NBD) photoswitch is a [2 + 2] cycloaddition to the strained higher energy quadricyclane (QC) system.

Multiphotochromic molecular systems have been reviewed in general in 2015,^17^ as well as from an application-based point of view with a particular focus on data storage,^18^ and more specifically photo/thermochromic macrocyles^19^ and multi-azoarene systems.^20^ No reviews thus far have reported specifically upon multiphotochromic NBD/QC photoswitches, and many advances in the area of energy storage have also been made in the field since the topic was first reviewed. This review is therefore focused on multiphotochromic molecular systems based on the molecular systems presented in Scheme 1 with an emphasis on energy storage applications.

## Multichromophoric systems for energy storage

Various parameters are necessary to characterise a photoswitch. The optical absorption, required to excite parent A to A*, must be red shifted so that it overlaps with the solar spectrum. This excited state then converts to the high-energy meta-stable photoisomer state B with a quantum yield of isomerisation defined as the number of converted molecules divided by the number of photons absorbed (per unit). Triggering the back-reaction by either thermal activation, a catalytic system, or light, should result in a release of energy as heat, corresponding to the energy storage (J mol^−1^), Δ*G*_storage_. Considering the molecular weight of the system, this value can be reported in terms of kJ kg^−1^, giving an energy storage density. The barrier for the back-conversion of B to A is related to the storage half-life (*t*_1/2_) of the system, which should be high enough for energy storage purposes over long periods of time, *i.e.*, on the timescale of days.

**Fig. 1:**
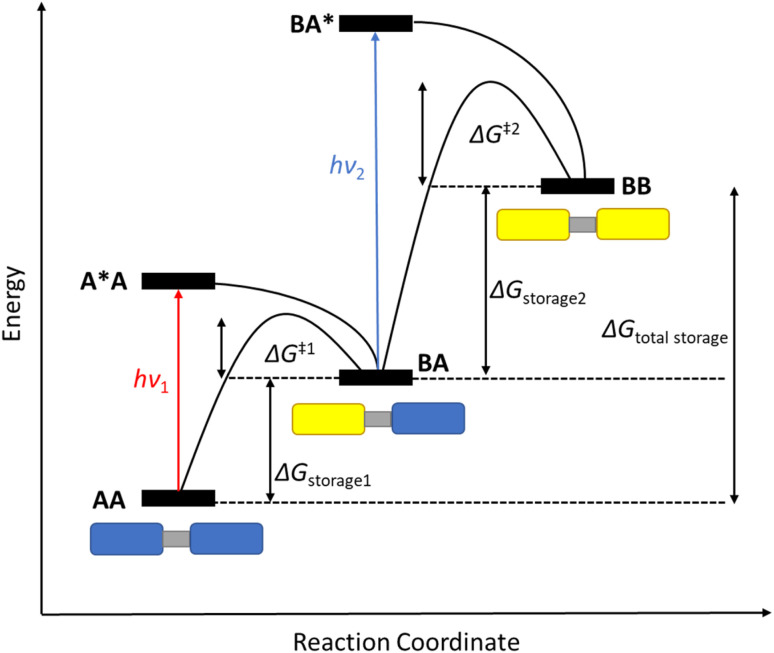
Schematic energy level diagram for the stepwise photoisomerisation of a photochromic dimer AA (blue rectangles) to its photoisomer BB (yellow rectangles) through the mono-switched BA. Note: drawn with a blue-shifted absorption after the first conversion, as seen for negative photochromic molecules such as NBD^13^ (figure adapted from ref. 13).

In theory, the more photochromic units that are added to a system, the more energy can be stored in the form of their photoisomers; if the number of photoswitchable units is *n*, the total number of possible states is equal to *n* + 1. The energy level diagram shown in Fig. 1 highlights this for a dimeric system where AA can be converted into its photoisomer BB through the mono-isomerisation product BA. The energy level diagram can vary slightly depending upon the nature of the photoswitch and the relative energies of absorption of each photoisomer, as well as the barrier for the back conversion process. Fig. 1 depicts a system where the absorption is blue-shifted after the first conversion step (*hν*_1_ < *hν*_2_), as is observed for negative photochromic molecules such as NBD, meaning there is a higher energy of isomerisation after the first conversion, which may cause problems with the second. For positive photochromic molecules such as azobenzene and DHA, *hν*_2_ < *hν*_1_.

Many factors must be considered when designing multichromophoric photoswitches, for example avoidance of the competing back reaction. The half-life time (*t*_1/2_) of each photoproduct BA and BB will depend on the energy barrier of each thermal back reaction, namely Δ*G*^‡^, and any competing absorption. The relative energies of each back reaction are also important as, for example, in Fig. 1, Δ*G*^‡2^ > Δ*G*^‡1^ such that when the first back reaction is triggered, full discharge of the system will be achieved, as observed for some NBD oligomers.^13^ The energy barrier of the second thermal back reaction can be larger, for example in some macrocyclic DHA systems^21^ which will be discussed later, meaning that the first discharge is fast, followed by a much slower event. All these factors govern the storage times of the higher energy metastable photoisomers as well as their potential applicability in MOST systems.

### Norbornadiene/quadricyclane

Several studies have been reported on the design and photochemical properties of the norbornadiene/quadricyclane couple (NBD/QC) for MOST applications.^2^ Unsubstituted NBD, the ‘parent’ compound, has an impressive energy storage density of 1 MJ kg^−1^ due to the highly strained nature of QC, though NBD absorbs only in the UV region of the electromagnetic spectrum with an onset of absorption of 300 nm.^22^ Design strategies for red-shifting the absorption and hence improving the energy storage properties of NBDs by providing a better match with the solar spectrum have been well established.^23,24^ These include incorporating either donor (D) groups on one double bond and acceptor (A) units on the other,^5^ or a D and A group on the same double bond.^2,25^ Similar strategies can be applied when designing multichromophoric photoswitches.

**Fig. 2:**
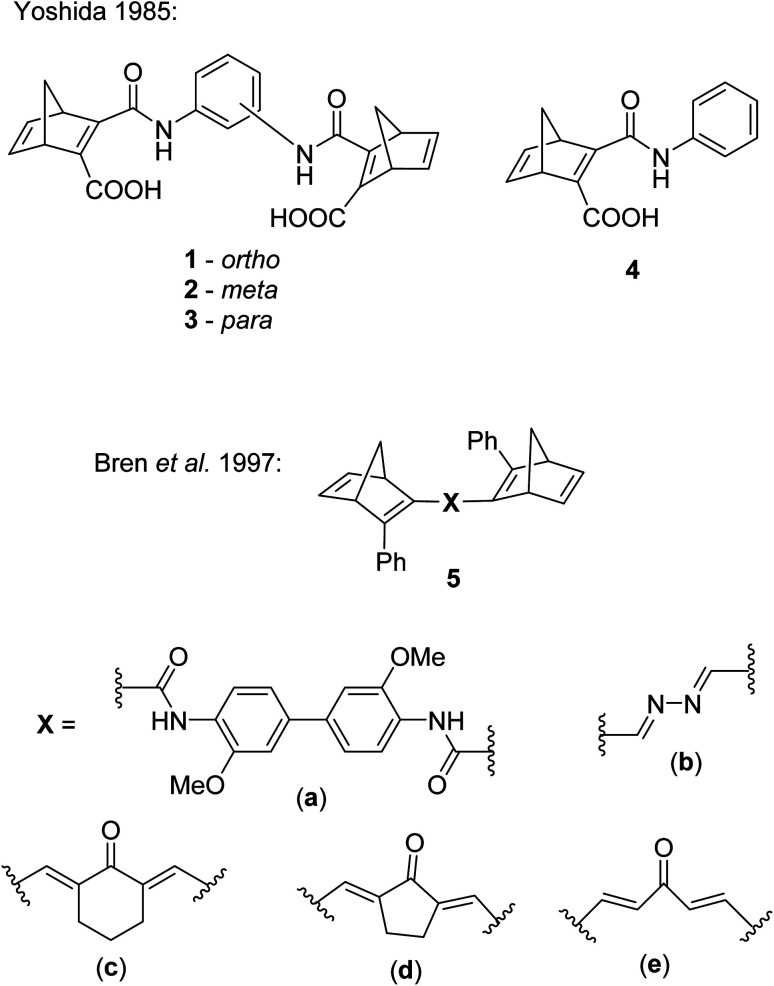
Biphotochromic systems 1–3 reported by Yoshida in 1985,^5^ along with the mono-analogue 4 for comparison, and 5a–e by Bren *et al.* in 1997.^26^

The first examples of NBD dimers were reported by Yoshida in 1985,^5^ comprising of two NBD units linked *via* a phenyl ring (either *ortho*-, *meta*-, or *para*-substituted) with an amide bond (Fig. 2). These systems are completed by a carboxylic acid group on the same double bond of each NBD. The dimers 1, 2 and 3 showed absorption onset wavelengths of 410, 420 and 460 nm respectively, red-shifted with respect to the mono-analogue 4 (*λ*_onset_ = 396 nm). The dimers also suffered from low photoisomerisation quantum yields with a maximum of 0.03, particularly for the *para*-substituted compound 3 (QY = 0.0004), as expected from the red-shifted absorption; a red-shift in absorbance is usually accompanied by a decrease in the QY, a challenge that needs to be overcome in the design of viable MOST systems. Even the mono-analogue 4 suffered a low QY of 0.09. No UV-vis spectroscopy photoconversion studies were conducted on these dimers, likely due to the low QYs, therefore no conclusions could be drawn about achieving the multiple possible states of these systems.

**Scheme 2:**
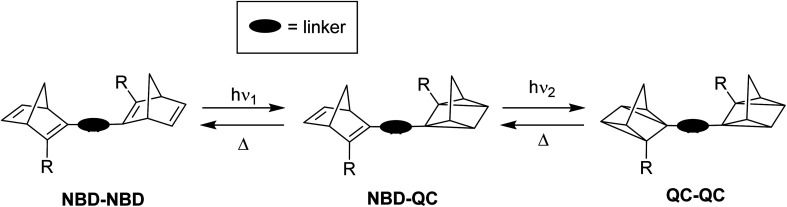
Illustration of the stepwise conversion of NBD dimers.

Some other early examples of biphotochromic NBD systems were reported by Bren *et al.* in 1997, featuring two NBD units bonded by various linkers (Fig. 2).^26^ UV-vis spectroscopy was used to first confirm the presence of the mono-quadricyclane due to the emergence of a new absorption band at 380 nm, as well as a band at *λ*_max_ = 300 nm upon prolonged irradiation at 365 nm, indicative of the bis-quadricyclane photoproduct. Despite these compounds also suffering from low QYs with a maximum of 0.04, promise is shown in the red-shifted absorption profiles (with absorption onset values of 435, 475, 530, 560 and 530 nm for 5a–e respectively), as well as proof of the ability to selectively target each state (Scheme 2). It is worth noting that different research groups have reported absorption onset wavelengths (*λ*_onset_) slightly differently, for example for compounds 1–4, *λ*_onset_ is defined at *ε* = 35, whilst for 5a–e it is when *ε* ≈ 0. Another important factor to consider with regards to energy storage applications is not only the *λ*_onset_ of the NBD–NBD compound, but also the difference in *λ*_onset_ when compared to that of the QC–QC photoproduct (the absorption/spectral window); as the wavelength difference decreases, the QY tends to decrease as there is more opportunity for competitive absorption to promote the back reaction. This trend is roughly followed with compounds 5a–d (Table 1).

**Table 1:** Absorption characteristics and photoisomerization quantum yields for 5a–e^26^

Compound	*λ*_onset NBD–NBD_/nm	*λ*_onset QC–QC_/nm	Spectral window^a^/nm	QY^b^
5a	435	400	35	0.011
5b	475	420	55	0.025
5c	530	455	75	0.039
5d	560	470	90	0.022
5e	530	500	30	—

a The spectral window is the difference in the absorption onsets for the NBD–NBD and QC–QC isomers. Data is recorded in 2-propanol with irradiation with filtered 365 nm (1a and b), or 436 nm (1c–e) light of high-pressure mercury lamp.

b QY for full conversion of NBD–NBD to QC–QC.

**Fig. 3:**
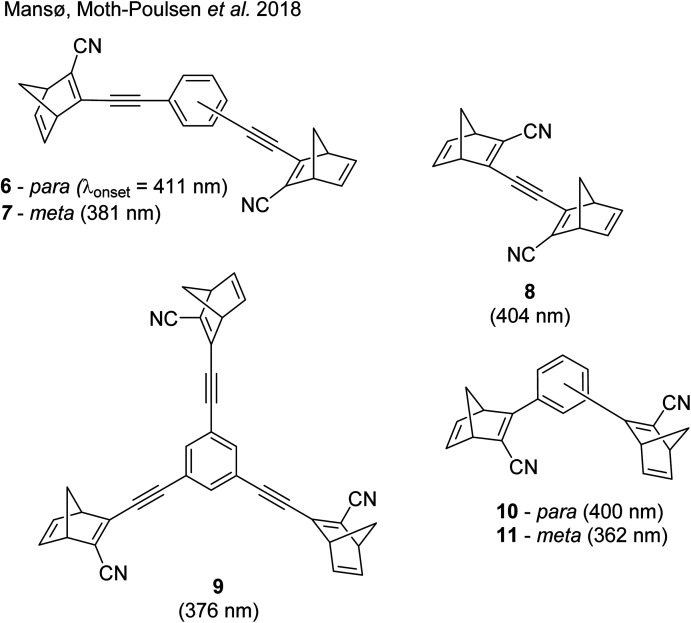
Structure of the oligomeric NBD units reported by Mansø, Moth-Poulsen *et al.*, with *λ*_onset_ values given in parentheses.^13^

These early examples of NBD dimers reported by Yoshida and Bren,^5,26^ are in essence not conjugated across the full system, meaning there is little or no electronic communication between each NBD unit. Providing a conjugation pathway across the whole molecule to connect the photoswitch units can drastically alter the properties of such systems and allow the possible benefits to be harnessed. More recently in 2018, Mansø and Moth-Poulsen *et al.* reported fully conjugated NBD dimer and trimer systems,^13^ with a bridging group that acts as the donor, and acceptor cyano groups completing the structure (Fig. 3). Cyano acceptors have been widely studied in mono-NBD derivatives due to their low molecular weight and ability to red shift the absorption profile.^27^ In this study, compounds with both *para* and *meta* linkages were synthesised, either with (6 and 7) or without (10 and 11) an acetylene linker to the phenyl bridging unit, the latter studied in an aim to reduce the molecular weight to improve the overall energy storage densities. A trimer (compound 9, Fig. 3) was also studied, that should have four overall states upon switching of each individual NBD unit to QC (NBD–NBD–NBD, NBD–NBD–QC, NBD–QC–QC, and QC–QC–QC). Compounds 6, 7 and 9, as well as 8, a derivative with only an acetylene bridging unit, could be synthesised *via* Sonogashira cross-coupling reactions, whilst 10 and 11 were targeted from a Diels–Alder reaction between either the *para* or *meta* bis(cyanoethynyl)benzenes and cyclopentadiene. The latter route is perhaps more attractive for MOST applications as it has been proven to be scalable with flow chemistry techniques.^28,29^ It should be noted that for NBD dimers, there exists the possibility of diastereoisomers, a fact that is often neglected; it was noted in this study that all compounds were isolated as a mixture of diastereoisomers though only one is shown in each case.

**Fig. 4:**
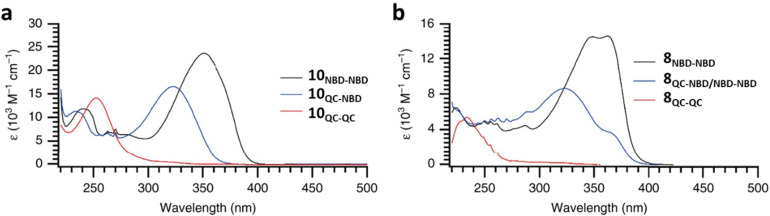
UV-vis spectra for the stepwise conversion of dimers: (a) 10 and its isomers; (b) 8 and its isomers, including the thermal–photostationary state between 8 and 8_QC–NBD_.^13^ Reproduced according to Creative Commons CC BY license.

As shown by Yoshida and Bren above, there is a large difference in the absorption onset for *meta* and *para* isomers, with the *para* isomers displaying a more red-shifted profile as expected due to the extended linear conjugation pathway between the two NBD chromophores (absorption onset values are given in Fig. 3). Upon irradiation close to the absorbance maxima (365 or 340 nm), the dimers and trimer could all be fully converted to the corresponding QC–QC compounds. Switching to the intermediate state QC–NBD could only be achieved for 8 and 10 by irradiation at 405 nm, though for compound 8 a thermal–photostationary state (PSS) was formed between 8_NBD–NBD_ and 8_QC–NBD_, dependent upon the intensity of the lamp and the temperature of the sample, due to fast back conversion of 8_QC–NBD_ (Fig. 4b). NMR studies were used alongside UV-vis spectroscopy to confirm the presence and ratio of each isomer state. It was ultimately possible to achieve the state 10_QC–QC_ upon irradiation at 405 nm though this took over 40 h; the absorbance of 10_QC–NBD_ at 405 nm is close to non-existent, thereby giving a spectral window for the sequential switching (Fig. 4a).

QYs were calculated based on the number of photoconversion events, taking into account that two or three NBD subunits are present per oligomer, with the assumption that each NBD unit is behaving as a separate entity with a similar absorption profile. For 8 and 10, which exhibit sequential switching, it was possible to measure the photoisomerization quantum yield at different wavelengths to independently probe the conversion of the two consecutive isomerization processes, though for 8_NBD–NBD_ only the first photoconversion QY could be measured due to the fast rate of back-conversion. The measured QYs are near quantitative, ranging from 0.53 for 11 up to 0.94 per NBD subunit for compound 6.

The UV-vis spectra displayed in Fig. 4 for 8 and 10 highlight the blue-shift after each photoisomerization event, an attractive feature which furnishes a very energetic QC–QC state, meaning the possible energy storage potentials are higher, as well as giving prolonged storage times. The half-life times, *t*_1/2_, of the QC–QC states range from 4.33 hours for 6, up to 48.5 days for 11, with the first energy discharge being the rate-determining step, attributed to the coupled nature of the two different electronic systems. This means that the energy level diagram in Fig. 1 holds, and full discharge of the system could be achieved upon triggering the first energy discharge event, *i.e.* QC–QC to QC–NBD. This is in contrast to DHA/VHF and other systems that will be discussed in the following sections,^30^ establishing NBD dimers as attractive candidates for energy storage applications. The calculated energy densities of the dimer and trimer systems are up to 927 kJ kg^−1^ (257 W h kg^−1^) with measured densities (heat release, Δ*H*) of up to 559 kJ kg^−1^ (155 W h kg^−1^) (Fig. 26). It is important to note that it is the amount of energy stored per kilogram not per mole that is important for applications, hence why we must consider the molecular weight of the photoswitch, and why dimers/oligomers provide an interesting alternative to bypass this problem somewhat due to the ‘shared’ molecular weight between multiple NBD units.

**Fig. 5:**
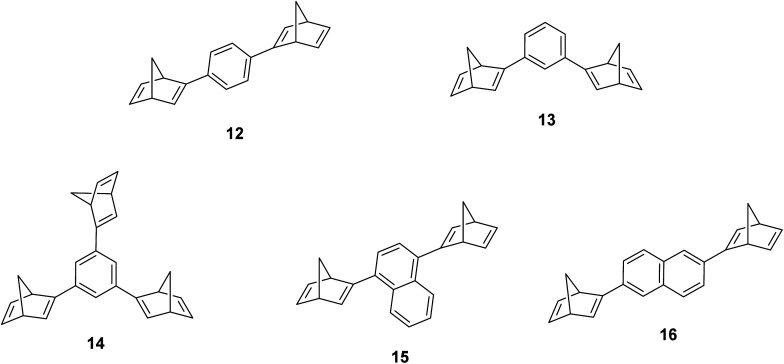
Bis- and tris-norbornadienes reported by Schulte *et al.* featuring only one substituent on each NBD unit to improve the energy densities by decreasing the molecular weight.^31^

Schulte *et al.* studied a series of similar bis- and tris-norbornadienes (Fig. 5) linked with either aryl or naphthyl rings.^31^ A key difference in the structure of these NBD oligomers is that there is only one substituent on the NBD double bond as opposed to the previously discussed structures that feature a donor–acceptor design. This led to higher recorded energy densities (Fig. 26), in particular for compound 14 at 734 kJ kg^−1^, due to the lower molecular weight of the systems, as well as high storage half-lives of up to 14 days. However, since these systems bear no donor–acceptor units, the absorption is in the range of 332–386 nm compared to 362–411 nm for those with the cyano substituents reported by Moth-Poulsen *et al.*^13^ The most red-shifted absorption is seen for compound 16 due to the extended conjugation provided by the naphthyl unit, though this also leads to the lowest observed QY of 0.08. The trimer compound 14, with the highest recorded energy density, exhibits a QY of 0.54 per photoisomerization event, comparable to that of the dimer compound 11 discussed above that showed the highest recorded energy density of 559.3 kJ kg^−1^, though 11 had a longer storage time with a half-life of 48.5 days compared to 8 days for 14. The energy densities per mol of compound are 172.5 kJ mol^−1^ and 255.8 kJ mol^−1^ for 11 and 14 respectively. As 11 is a dimer and 14 is a trimer, one can compare the efficiency of each photochromic unit by dividing the energy density by two or three, which gives a similar energy storage per unit (roughly 85 kJ mol^−1^). Nevertheless, 14 has the higher recorded energy storage density per kg of compound which is more important for MOST applications.

Real-time monitoring of the photoisomerization process was also performed using *in situ* NMR spectroscopy for the first time. The *para*-substituted derivative 12 could be converted quantitatively to the corresponding bis-QC, though irradiation of the other bis-substituted NBDs resulted in photostationary states (PSS) that still contained the bis- and mono-norbornadienyl-substituted components as well as the fully converted product in ratios of 5/24/71, 12/34/54, and 12/29/59 for 13, 15, and 16 respectively, where the ratios are given in the order of increasing QC units. Likewise, the PSS of the tris-NBD (14) consisted of the unconverted, mono-, bis- and tris-converted products in a ratio of 3/4/9/84. If the PSS could be driven towards the fully converted QC compound, the energy storage density could be improved even further. Cyclability studies were also performed to test the stability of these compounds, showing an average degradation of 1.53% per conversion over the 10 conversions studied. These values are slightly higher than those of 6 and 10 which showed decomposition values of 0.16% and 0.11% per cycle over 71 cycles. These values indicate that these NBD dimers and trimers show promise as candidates for MOST applications.

**Fig. 6:**
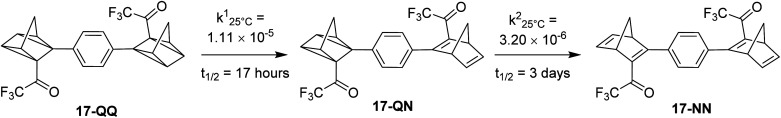
Trifluoroacetyl-substituted NBD dimers reported by Petersen *et al.* in 2019 highlighting the back reaction processes from QC–QC to NBD–NBD; the first back conversion is quicker than the second.^25^

Petersen *et al.* reported a similar structure to 10, but with trifluoroacetyl acceptors in place of cyano, namely compound 17 (Fig. 6).^25^ A red-shift in the absorption spectrum was observed for 17 compared to 10 (*λ*_onset_ = 466 and 400 nm respectively) owing to the stronger electron-withdrawing ability of the COCF_3_ groups compared to CN. As discussed previously, compound 10 displayed stepwise photoconversions upon irradiation at 405 nm, with identification of the NBD–QC intermediate. In comparison, 17 does not show any sequential photoswitching events of the forward reaction, though stepwise kinetics were observed for the back-conversion from the QC–QC form. The first back-conversion (QQ-17 to QN-17) was significantly faster than the second (QN-17 to NN-17), opposite to what was observed for systems 6–11. The QYs for the stepwise photoconversions were measured at both 340 and 405 nm, yielding comparable results of 0.77 at both wavelengths, slightly superior to that of 10 which had sequential quantum yields of 0.73 for the first photoconversion and 0.51 for the second. In this study, compound 17 was also incorporated into a polystyrene (PS) matrix to assess the possible applications in window laminating for climate control. The measured energy density, Δ*H*_storage_, for a 10.8% (by wt) loading of the NBD dimer in PS came out at 51.8 kJ kg^−1^.

**Fig. 7:**
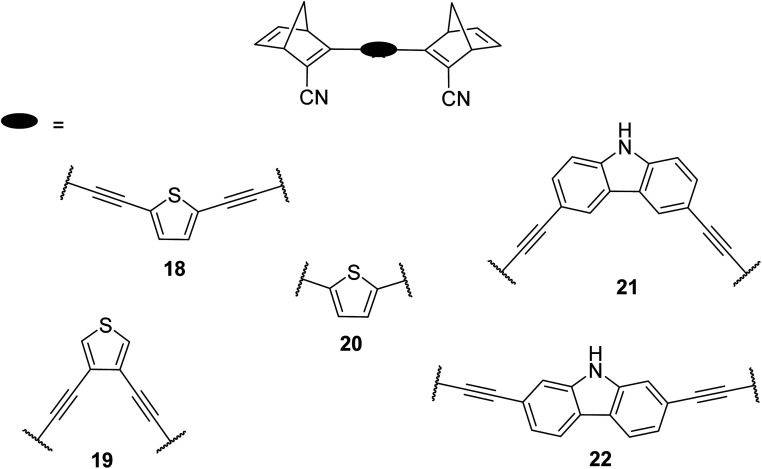
Structure of dimers linked by various heterocycles reported by Mansø and Nielsen *et al.*^32^

Similar dimer structures but incorporating heteroaryl electron-donating linkers, either thiophene or carbazole, were reported by Mansø and Nielsen *et al.* (Fig. 7).^32^ The most impressive result is that the absorption onsets for the linearly conjugated compounds with the thiophene-2,5-diyl bridge are red-shifted by 57 and 49 nm (to 468 and 460 nm for 18 and 20 respectively) compared to the *para*-phenylene linked dimer discussed above (411 nm for 6). These absorption onsets are also hugely red-shifted compared to the cross-conjugated thiophene-3,4-diyl substituted compound 19 (381 nm), a trend also observed for the phenylene-bridged compounds 6–11. The connectivity influences the absorption onset for the carbazole isomers too, though 22 is only 12 nm further red-shifted than 21 (with values of 432 and 420 nm for 22 and 21 respectively). Interestingly, both the absorption onset and maximum are similar for the directly linked dimer 20 and the alkyne-linked dimer 18 (460 and 468 nm), despite a shorter linear conjugation pathway in the former. Furthermore, the spectral window (the difference in *λ*_onset_ between NBD–NBD and QC–QC) is an impressive 134 nm for 20, the largest in the series.

The photoisomerisation QYs were calculated in the same way as discussed for others, taking into account that there are two NBD subunits present in each of the dimeric structures, hence the QY is per NBD subunit. The values were determined to be 0.5, 0.44 and 0.7 for compounds 19, 21 and 22 respectively. It was not possible to measure the QY for 18 and 20 due to markedly different QYs for the two isomerizations leading to non-linearity in the measurements. Half-lives were determined by measuring the kinetics of the back-reaction for at least three different temperatures, whereafter the half-life at 25 °C could be extrapolated using the Arrhenius equation. The half-lives for all the compounds are relatively similar to the phenylene-bridged compounds reported by Mansø and Moth-Poulsen *et al.*^13^ The substitution pattern of the thiophene has a strong influence on the reaction profile; 3,4-substitution (compound 19) increases the half-life of the QC–QC state compared to the 2,5-substituted compound 18 (16.1 and 1.32 hours for 19 and 18 respectively), a trend observed for the *meta*- and *para*-phenylene bridged compounds discussed above. Removing the alkyne linkers drastically reduces the half-life to only 43.8 seconds for compound 20, rendering this compound not viable for MOST applications. The carbazole systems also displayed shorter half-lives than the thiophene-linked compounds, and thus seem less suitable for MOST systems and more suitable for other fields such as photopharmacology applications.^33^

**Fig. 8:**
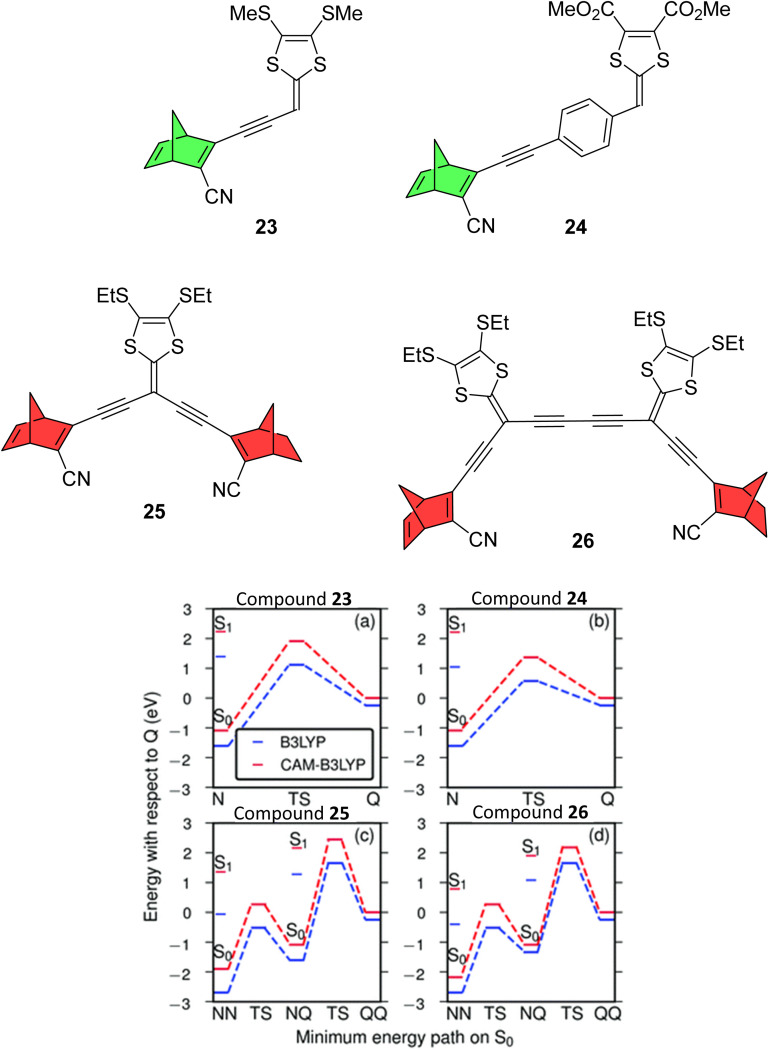
Top: Dithiafulvene-substituted NBD dimers and respective mono-substituted NBDs reported by Mansø *et al.*^34^ Note that the mono-substituted NBDs exhibited photoswitching (green) whereas the dimers did not (red). Bottom: Energy landscapes from TDDFT using B3LYP (blue) and CAM-B3LYP (red). Reproduced from ref. 34 with permission from the PCCP Owner Societies.

A follow up study by Mansø *et al.* reported dithiafulvene (DTF) linked NBD dimers (Fig. 8),^34^ a strategy employed as DTF is a good electron donor due to its low oxidation potential. DTF and tetrathiafulvene (TTF) have been added to DHA photoswitches in the past to successfully induce a red shift in the absorption profile.^35,36^ All compounds reported in this study showed impressive red-shifted absorption profiles compared to unsubstituted NBD (*λ*_onset_ = 509, 499, 556 and 548 nm for 23–26 respectively), with the dimers (25 and 26) absorbing further into the red than analogous mono-substituted NBD compounds (23 and 24). However unfortunately, UV-vis studies of the photoconversion of the dimers showed no change upon irradiation at 340, 357, 429 and 474 nm for 25 and 341, 408 and 474 nm for 26. The mono-NBD compounds 23 and 24 could be switched, though for 23 no isosbestic points were observed for the thermal back-reaction, likely suggesting degradation. Compound 24 showed an equally slow NBD-to-QC photoisomerization process as for 23, requiring continuous irradiation for 8 hours to achieve full isomerization. Oxidative dimerization is known to occur for dithiafulvenes, which may be responsible for the degradation of these compounds, if oxidation of 24 to its reactive radical cation is possibly promoted photochemically.

To rationalize the experimental results, computational studies were performed. In order for a photoswitch to work, the S_1_ energy for the initial state must be higher than for the transition state (TS). By mapping out ground and excited energy landscapes using static and time-dependent DFT calculations, it was shown that 23 and 24 fulfil these basic energy conditions, whereas for the dimers 25 and 26, the situation is more involved. Whilst the energy condition is fulfilled for the first isomerisation step (NBD–NBD to NBD–QC), the S_1_ energy of the intermediate state (NBD–QC) is much lower than the energy of the second TS. Moreover, the lowest energy absorption for NBD–QC is similar to that for NBD–NBD, suggesting that one of the two NBD units in 25 and 26 can be switched, but photo-absorption of NBD–QC creates an excited state that cannot overcome the barrier to the fully switched QC–QC configuration, so instead relaxes back to the initial NBD–NBD state. To put this into context with respect to the phenylene-linked compounds 6–11, due to the significantly red-shifted absorption onsets for 25 and 26, the entire S_1_ landscape should be shifted notably downward, which pushes the S_1_ energy for the intermediate NBD–QC configurations below the TS for the NBD–QC to NBD–NBD conversion. These results highlight the difficulties in designing multistate photoswitches to ensure that each photochromic unit is addressable, whilst simultaneously trying to redshift the absorption profile.

**Fig. 9:**
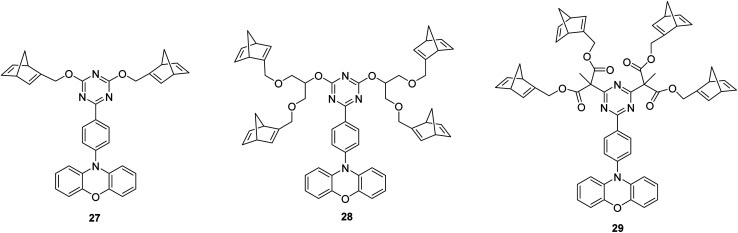
NBD oligomers featuring a thermally activated delayed fluorescence (TADF) molecule core.^43^

An alternative strategy to redshift the absorption wavelength of NBD to match that of the sunlight reaching the earth is to utilise a photosensitizer^37^ whose absorption spectral profile matches the solar radiation.^38–42^ A unique approach to NBD dimers was reported by Chou and co-workers^43^ whereby thermally activated delayed fluorescence (TADF) molecules were exploited as photosensitizers to optimise the thermal energy storage, whilst simultaneously providing a means to monitor the photoisomerization process. Here a phenoxazine–triphenyltriazine (PXZ–TRZ) core is anchored with two or four NBD units to give compounds 27, and 28–29 respectively (Fig. 9). PXZ–TRZ has been reported to exhibit prominent TADF, with T_1_ energies of ∼264–272 kJ mol^−1^ estimated from the onset of phosphorescence (448–455 nm) and a near-zero Δ*E*_S–T_ due to effective separation of the highest occupied molecular orbital (HOMO) and lowest unoccupied molecular orbital (LUMO) in a single molecule.^44^ With the photosensitizer being chemically linked to the NBD units, efficient intramolecular energy transfer can occur to populate the NBD triplet state, followed by NBD-to-QC conversion. The development of photosensitizers to optimize MOST systems is challenging in order to optimise the triplet state energy so that it is higher than that of NBD as well as possessing allowed electronic transitions similar to those of solar photons. To meet these requirements, the energy between the S_1_ and T_1_ states must be as close as possible, hence why TADF molecules were chosen as they possess spatially separated HOMO and LUMOs and a small energy difference, Δ*E*_S–T_, between the first excited singlet and triplet states.^45^

**Fig. 10:**
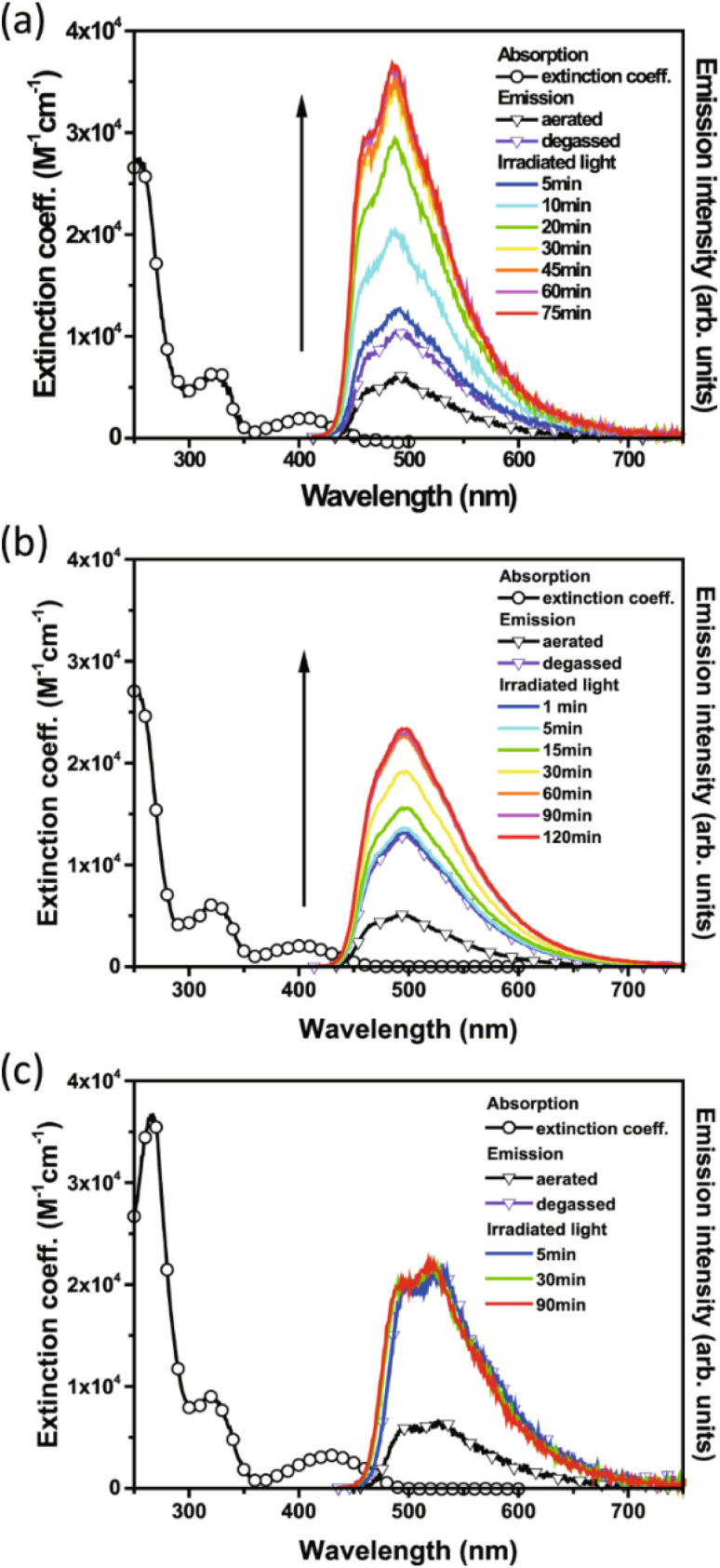
Absorption and emission spectra of 27 (a), 28 (b), and 29 (c) measured in cyclohexane (*λ*_e*x*_ = 405 nm).^43^ The arrow represents increasing emission intensity with photolysis time. Reproduced according to Creative Commons CC BY license.

Of the three compounds studied, 27 and 28 could be switched to their QC forms (confirmed by ^1^H-NMR spectroscopy) whilst 29 could not be switched. However, no intermediate species where, *e.g.*, only one QC is formed could be confirmed by ^1^H-NMR spectroscopy plausibly due to its short lifespan. The switchable compounds 27 and 28 exhibit TADF that is strongly quenched by energy transfer and is thus dependent on the NBD-to-QC conversion time; TADF intensity and dynamics can be used as a tool to monitor the photoisomerization process (Fig. 10). The lowest lying transition for the title compounds (S_0_ → S_1_) possesses charge transfer character, where the HOMO and LUMO are mainly located on the phenoxazine and triazine moiety respectively, with no localisation on the NBD units. This accounts for the similarity of the lowest energy absorption band for 27–29 (Fig. 10). Moreover, this supports the idea that the conversion is associated with the photoinduced energy transfer from the PXZ–TRZ core to the NBD units. The dynamics of the delayed fluorescence vary significantly as a function of irradiation time. The decay of 27 is initially fast at 0.7 μs before irradiation, indicative of quenching dynamics that can be well explained by intramolecular triplet (TRZ) to triplet (NBD) energy transfer. Irradiation at 405 nm leads to a gradual increase in the delayed fluorescence decay time, accompanied by an increase in the steady-state TADF intensity (see Fig. 10), until it reaches its maximum after approx. 90 min of irradiation (10.8 μs) and then remains unchanged, indicating complete conversion. Similar dynamics were also observed for 28, whilst 29 shows an almost constant TADF rate upon continuous irradiation, indicative of the retardation of triplet–triplet energy transfer and hence a lack of NBD-to-QC isomerization. This was further verified by simulation results, where the rate of triplet–triplet energy transfer in 29 is significantly lower than other competing rates by approximately two orders of magnitude, due to 29 having a lower T_1_ energy than NBD. Upon catalysis, the reverse QC-to-NBD reaction occurred at room temperature, converting the stored chemical energy back to heat with excellent reversibility; the storage capacity for 27 was determined to be 280 kJ kg^−1^.

### Azobenzene

Azobenzene derivatives have shown great potential as active MOST candidates.^46,47^ Moth-Poulsen and co-workers demonstrated the first operating lab scale experiment including solar energy capture/storage and release with a liquid mono-azobenzene.^48^ Despite the compound showing promising photochemical parameters such as a long thermal half-life of 36.3 hours and strong absorption at 350 nm, the estimated maximum storage efficiency in the neat state was calculated to be 0.88%, much less than those achieved with NBD derivatives. Li and co-workers recently reported a systematic evaluation of the solar efficiency of azo-switches and found them all to be below 1.0%.^49^ Further improvement to increase the quantum yields and diminish absorption of the *Z*-photoisomer, as well as differentiate the spectral difference between *Z* and *E* states, would be required in order for azobenzenes to become more viable candidates for solar thermal energy storage applications. Nevertheless, azobenzenes have unique properties over other photoswitches, in particular they can also show phase changes.^50–52^ Incorporating two or more azobenzenes into the same molecule to form multi-state photoswitches may offer an interesting strategy to optimise the properties for solar thermal energy storage.^53^

Multichromophoric photoswitches featuring various azoarene derivatives have been reviewed in detail elsewhere,^20,46,54^ though here we will focus specifically on the potential of multistate azobenzene photoswitches for energy storage applications. In general, they can be classified according to their structure as linear, non-linear or bent,^55^ branched,^56^ tripodal *C*_3_ symmetric,^57^ and macrocyclic,^58–60^ where the design of such systems is crucial in determining their properties. For example, linear *ortho*-linked multi-azobenzenes do not show any *E*-to-*Z* isomerization due to the inhibition of photochromism by *ortho*-substitution, or if they do, the rate of the *Z*-to-*E* thermal back reaction is on the order of milliseconds so must be observed by ultrafast transient absorption and flash photolysis experiments.^61^ Linear *meta*-linked multi-azobenzenes behave similarly to multiple independent azobenzenes, with their absorption spectra resembling the sum of the spectra of the constituent azobenzene units with no shift in the absorption maxima. Finally, linear *para*-linked multi-azobenzenes exhibit a regular red shift and increase in the molar extinction coefficient upon increasing the number of azobenzene groups, though they suffer a decreased photoreactivity (lower excited state lifetime and low isomerization QY) due to strong electronic communication amongst the azobenzene groups. Several approaches of breaking the conjugation between *para*-bisazobenzenes have been presented,^62^ though in order to achieve multistate switching, differently substituted azobenzene units must be incorporated, resulting in more complex designs; multiphotochromic azobenzene compounds that can be addressed selectively are still exceptional.^63^

There is various emerging research into azobenzene-grafted polymers, azobenzene-functionalised carbon nanomaterials,^7,64,65^ and also the use of nanoparticles^66,67^ to enhance solar thermal energy storage. Kolpak *et al.* used DFT to investigate different carbon-based templates in combination with various azobenzene photoswitches as potential chromophore/nanostructure hybrids for customizable energy storage applications.^68^ They found that by using 1,3-bis(phenylazo)benzenes containing two azo units, the amount of energy that could theoretically be stored is doubled, with only a 33% increase in molecular weight, compared to the mono-azo counterpart. They demonstrated, through theoretical calculations, that these fuels can reversibly store solar energy at densities comparable to Li-ion batteries, with high thermal stability and efficiency of solar-to-heat conversion.

**Fig. 11:**
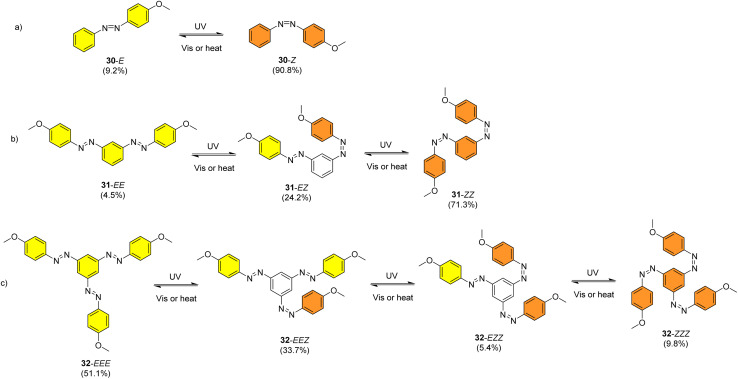
Mono-, bis- and trisazobenzenes studied by Sun *et al.* The value in brackets indicates the percentage of each state present upon charging with UV irradiation.^69^

Sun *et al.* studied the solar thermal energy storage of compounds 30–32 (Fig. 11) to investigate the structure–property relationship of azobenzenes for MOST applications.^69^ Though the identical monoazobenzene^70^30 and similar bis-^71^ and tris-azobenzenes^67^ (a study which will be discussed later) had been reported previously, their investigation was the first to reveal how the energy storage properties are related to the number of azo units in the molecule. The absorption spectrum of the π–π* transition bands of 30-*E*, 31-*EE*, and 32-*EEE* at the same concentration of 50 μM revealed a ratio of approximately 1 : 2 : 3, indicative of the azo branches in 31 and 32 being electronically decoupled from one another and essentially behaving like independent azobenzene molecules, due to their *meta* relationship. The QY values for the *E*-to-*Z* photoisomerization in solution at 365 nm were 0.23, 0.11 and 0.09 for 30, 31 and 32 respectively, within the scope of QYs for typical azobenzenes (0.05–0.21). The half-life values for 30, 31 and 32 decreased with increasing number of azochromophores, with values of 37.7, 28.1 and 20.5 h respectively. This phenomenon is opposite to that observed for the NBD/QC system where photoswitchable oligomers increased the storage time, as discussed previously (see compounds 6–11).^13^ The trend for these azobenzenes can be explained due to the existence of more twisted and metastable non-planar conformations in the multi-azobenzenes in the *Z* state, which causes the systems to be less stable in their *Z*-rich state as they have a stronger tendency to isomerize to their thermally stable *E* isomer. ^1^H-NMR spectroscopy was used to confirm the ratio of each state upon charging with UV irradiation (values given in Fig. 11), due to the characteristic OMe peak. The overall *Z*-isomer content for 30, 31 and 32 is 90.8%, 83.4% and 24.6% respectively, indicating a decrease in *Z*-isomer content with increasing number of azo-chromophores, following the trend in half-lives.

Energy storage studies demonstrated that 30, 31 and 32 can be charged in solution, with the bisazobenzene 31 showing the best energy storage performance with an energy density of 272 kJ kg^−1^ (Fig. 26). The tri-substituted compound 32 had a relatively low energy density, due to the large steric hindrance and low degree of isomerization. It is worth noting that the relationship between the number of azo chromophores and the energy storage performance differed compared to the NBD/QC system; a larger number of azo chromophores does not necessarily improve the energy storage density. In the solid-state, only the mono-azobenzene 30, which displays photoinduced reversible solid-to-liquid transitions, could be charged, with the crystalline structures of compounds 31 and 32 hindering solid-state photoisomerization.

**Fig. 12:**
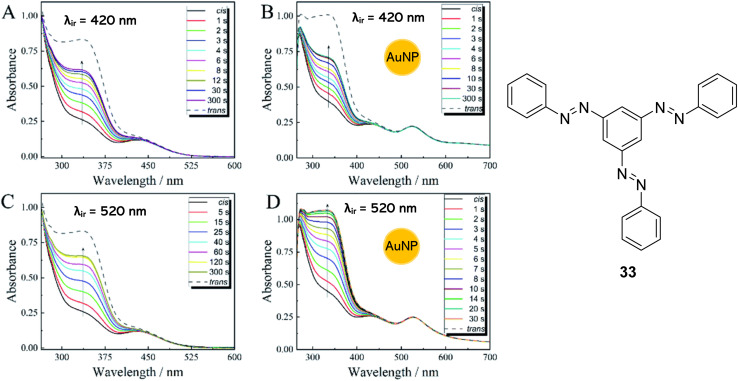
Time-resolved UV-vis absorption spectra of 33 (A and C) and 33 doped with AuNPs (B and D) at RT upon irradiation with 420 nm light (A and B) or 520 nm light (C and D), with the arrows indicating the order of the test.^67^ Graphs reproduced from ref. 67 with permission from the Royal Society of Chemistry.

Heating is the most frequently used strategy to overcome the energy barrier and release the stored energy in azo-based solar thermal fuels. However, heating is not desirable in practical STF applications, from an economic standpoint as well as the required heating limiting the potential heat output to only 2–15 °C. As mentioned above, light can be used to facilitate the back conversion, though this usually does not allow for complete back conversion into the parent *E*-state, a drawback of azobenzene photoswitches. Feng and co-workers^67^ presented the first example of a composite solar thermal energy storage material, polyvinyl alcohol (PVA) polymer-templated trisazobenzene compound 33 (Fig. 12), doped with gold nanoparticles (AuNPs) as a photothermal conversion material (PTM) to stimulate the discharging procedure by light and the thermal energy generated by the photothermal effect of the PTM. They showed that the stored energy of 33 could be released rapidly at RT with light as the only stimulus. Gold nanoparticles were chosen as they are well known to have extraordinary photothermal conversion capabilities due to their specific localized surface plasmon resonance effect.^72,73^ Moreover, monodisperse, spherical AuNPs have been proven to effectively accelerate the *cis*-to-*trans* isomerization of azobenzenes in the absence of light.^74^

Irradiation with light at 365 nm was effective for charging the PVA/33@AuNP STFs, whilst irradiation at 520 nm, which coincides with the plasmonic resonance of the AuNPs, induces the energy release from the samples. The energy densities of *cis*-33, measured using differential scanning calorimetry (DSC), were 111.1 kJ kg^−1^, 139.4 kJ kg^−1^ and 115.9 kJ kg^−1^ at heating rates of 1 °C min^−1^, 5 °C min^−1^ and 10 °C min^−1^ respectively. These moderate energy density values may be due to the *cis*-33 here being defined to include a mixture of (*E*,*E*,*Z*), (*E*,*Z*,*Z*) and (*Z*,*Z*,*Z*) isomers. Nevertheless, temperature increases of up to 8.8 °C were observed during the discharge of the PVA/33@AuNP STFs. The significant heat output is attributed to three cooperative effectives. Firstly, irradiation at 520 nm triggers the *cis*-to-*trans* isomerization of 33 whilst simultaneously stimulating the plasmonic photothermal effect in the AuNPs, releasing heat which in turn also stimulates the *cis*-to-*trans* isomerization. Finally, the AuNPs themselves can act as catalysts, accelerating the back isomerization process in darkness; the lifetime of the *cis*-33 is reduced from 568 h to 7 h upon the addition of the AuNPs. Trisazobenzenes themselves have long storage lifetimes due to the steric hindrance of the molecule which inhibits *cis*-to-*trans* isomerisation.^53^ Increasing the volume of AuNPs added to the trisazobenzene solution also increased the rate of the *cis*-to-*trans* isomerisation. Furthermore, the π–π* transition band intensity, characteristic of the *trans*-isomers, is completely restored for the AuNP-doped trisazobenzene upon irradiation at 520 nm within 30 s, whilst the band is never fully restored for irradiation of 33 alone (Fig. 12). Irradiation at 420 nm, however, has no noticeable effect on the back-conversion of the 33-AuNPs as this does not coincide with the plasmonic resonance of the AuNPs. The control of the lifetimes of these STFs with the addition of AuNPs of varying concentrations is useful in a variety of applications; STFs with longer lifetimes can be used as a stable heat resource, whilst shorter lifetimes benefit applications that require heat accumulation in a short time or within a small area.

**Fig. 13:**
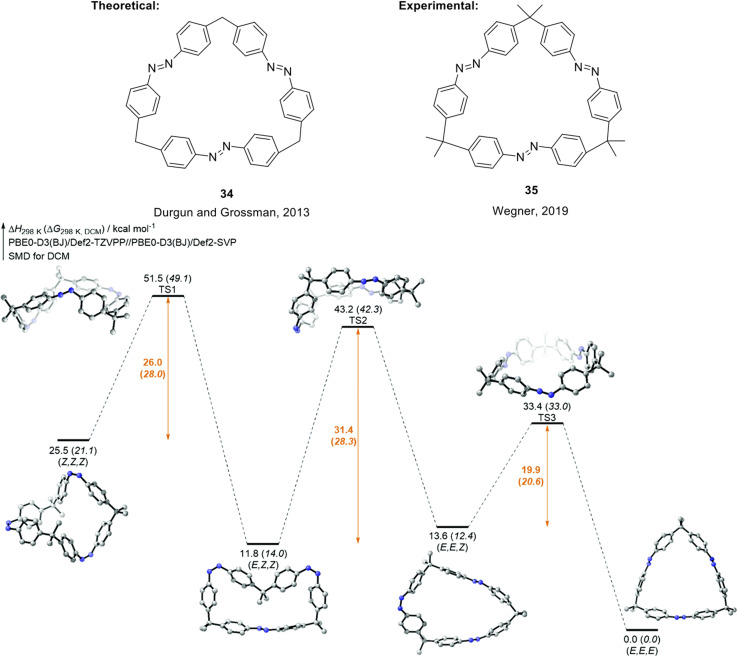
Structure of macrocyclic trisazobenzene 34 proposed by Durgun and Grossman to theoretically store 600 kJ kg^−1^ of energy,^59^ and structure of later synthesised macrocyclic trisazobenzene 35 with azobenzene units that can be selectively addressed.^63^ Bottom: Calculated enthalpy and Gibbs free energy pathway for 35. Reproduced from ref. 63 with permission from the Royal Society of Chemistry.

An alternative design strategy that can potentially improve the properties of azobenzene for MOST/STF applications has been proposed, and this involves introducing macrocyclic ring strain to give compounds called azobenzophanes.^75^ Computational modelling suggests molecular rings of linked molecules, for example compound 34 (Fig. 13), can improve energy densities due to the imposed strain in the molecules.^59^ Durgun and Grossman showed that by varying the linker group, number of azobenzene monomer units, and the position and number of hydroxyl groups on the azobenzene rings to induce H-bonding, a calculated maximum energy density of 600 kJ kg^−1^ could be feasible, as well as improving the stability of the fuels.^59^ Wegner and co-workers reported the synthesis and photochemical properties of a similar *tris*-azobenzene macrocycle differing to 34 only in the additional methyl groups in the alkyl backbone of the macrocycle, 35, which is capable of three-state isomerization (Fig. 13).^63^ Compound 35 can be selectively switched into the (*E*,*E*,*E*), (*E*,*Z*,*Z*) and (*Z*,*Z*,*Z*) states by light or light and heat as stimuli with more than 70% conversion efficiency. These properties were due to the remarkably high thermal stability of the (*E*,*Z*,*Z*) isomer, rationalised by a high (*E*,*Z*,*Z*) to (*E*,*E*,*Z*) activation barrier due to high ring strain that must be overcome in this step. The build-up of each isomer was confirmed by ^1^H-NMR spectroscopic studies, and the relative enthalpy and Gibbs free energy pathway of all the thermal isomerization steps was calculated.

**Fig. 14:**
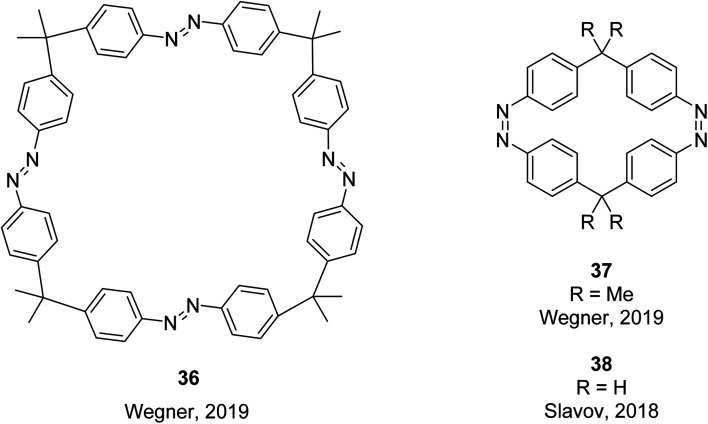
Thermodynamically stable configurations of the tetraazomacrocycle, 36,^63^ and bisazomacrocycles 37 and 38.^76^

The tetraazo-macrocycle 36, and bisazo-macrocycle 37, were also synthesised and studied (Fig. 14), and it was found that macrocyclic ring strain has a major influence on the isomerization behaviour. The internal sp^3^-carbon bridge angle, determined by X-ray crystallography from their molecular structures, gives an indication of the macrocyclic ring strain. The tetraazo-macrocycle 36 features an internal angle of 109.5°, close to the ideal tetrahedral angle, whilst the trisazo-macrocycle 35 deviates significantly from ideality with a smaller angle of 103°. Even the properties of the largest macrocycle 36 are influenced by ring strain. Again, three different PSSs were identified for 36, though each showed lower thermal stabilities compared to those of 35. In this case, the thermal (*E*,*Z*,*E*,*Z*) to (*E*,*Z*,*Z*,*Z*) isomerization was identified as the rate determining step where most of the ring strain is built up. In the case of the bisazobenzophane 37, the highly strained nature leads to the (*Z*,*Z*) isomer being the most thermodynamically stable. Isomerization of 37 did not occur photochemically or thermally within the timescale of the applied experiments. This was as expected based on previous studies, where continuous irradiation of a similar compound 38 did not lead to accumulation of the (*Z*,*E*) or (*E*,*E*) isomers, due to the ultrafast thermal back-isomerization pathway which prevents any photostationary accumulation of *E*-isomers.^76^ These results highlight there is a delicate balance when designing macrocyclic compounds, as ring strain has been computationally shown to improve energy storage densities, though too much can lock the compounds into one stable isomer, preventing any photoswitching from occurring. Despite compounds 35 and 36 being synthesised and studied for their selective photoswitching properties, the energy storage potential has not yet been recognised experimentally. The focus of macrocyclic azobenzenes tends to be more for host–guest supramolecular applications^77,78^ rather than energy storage. A similar strategy of introducing macrocyclic strain has also been employed in mono-hydrazone derivatives to improve the thermal half-life and energy storage properties of the molecules.^79,80^

**Fig. 15:**
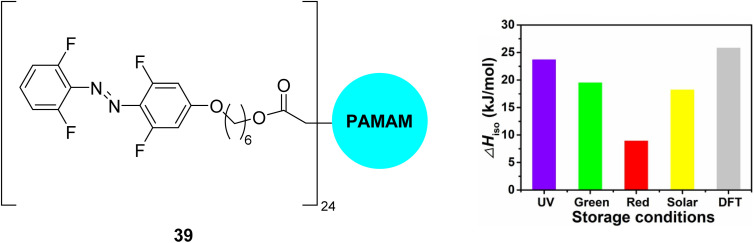
Azobenzene dendrimer 39 (where PAMAM is poly(amidoamine), a class of dendrimer made of repetitively branched subunits of amide and amine functionality) incorporated into wearable fabrics for solar thermal energy storage, reported by Li and co-workers.^84^ The graph shows energy storage densities (Δ*H*_iso_) for irradiation with different light sources as well as comparison to the calculated value from DFT. Graph reproduced from ref. 84 with permission from Elsevier.

Another approach to utilise the energy storage properties of multiphotochromic azobenzene systems is to use dendrimers or polymers of azobenzene.^81,82^ Combining MOST molecules with fabrics can be an effective strategy to tune the thermal performance of wearable fabrics,^70,83^ though these MOST/fabric composites often suffer limitations such as UV-dependent energy storage. Li and co-workers^84^ developed a flexible wearable fabric consisting of azobenzene-containing dendrimers 39 (Fig. 15), polydopamine (PDA), and cotton fabric, that can efficiently store UV, green, and red light, thereby having a good match with the solar spectrum, whilst also enabling applications in low- or room-temperature solvent-free environments. These properties are attributed to the red-shift of the n–π* band of the σ-fluoroazobenzene beyond 550 nm as well as good separation of the n–π* bands of the *trans* and *cis*-isomers, the low glass-transition temperature, *T*_g_, of the *cis*-isomers, and the photothermal effect of PDA. Storage energy densities of the wearable fabrics of up to 0.05 MJ kg^−1^ were achieved, comparable to those with solvent assistance and higher than those of the fabric without PDA, as well as storage half-lives of up to one month. The temperature of the wearable fabrics can also increase by 11.1–12.3 °C under blue light irradiation due to *cis*-to-*trans* isomerization and the photothermal effect, showing excellent results in room-temperature wrist guards and low-temperature body-warming applications.

### Dihydroazulene/vinylheptafulvene (DHA/VHF)

The dihydroazulene (DHA)/vinylheptafulvene (VHF) couple is a system that is photoactive in the forward direction and thermally active in the other, meaning a complete conversion between the two states is possible. Upon irradiation with light of approx. 350 nm, DHA undergoes a ten π-electron *retro*-electrocyclisation to form the higher energy meta-stable VHF, which in turn undergoes a thermally induced ring closure back to DHA. Compared to the NBD/QC photoswitch couple, calculations indicate that the energy density of the parent, unsubstituted DHA/VHA system (Scheme 1, R = ph) is only 0.11 MJ kg^−1^ (compared to 1 MJ kg^−1^ for unsubstituted NBD), with a half-life that is too short (approx. 3.5 hours) for practical energy-storage solutions.^9,85^ In order to render the DHA/VHF photoswitch system suitable for energy-storage applications, the energy density of the system must be increased, the absorption must be shifted from 350 nm towards the visible region, and control over the discharge event (*i.e.* back conversion from VHA to DHA) must be gained. Multiphotochromic systems may offer some solutions in these regards.^86^

**Fig. 16:**
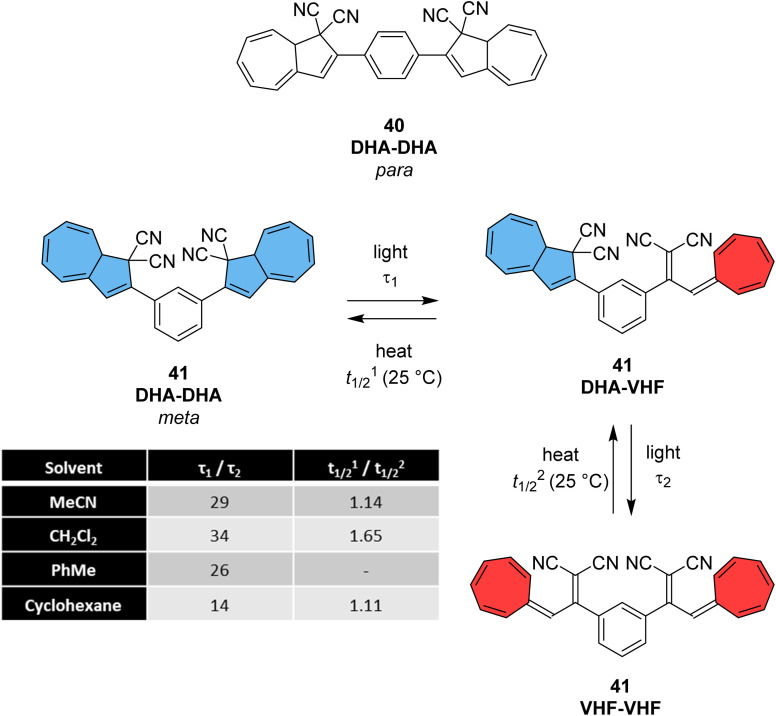
Top: *para*-Phenylene DHA dimer 40. Bottom: Light-induced ring openings of *meta*-phenylene DHA dimer 41 to VHF–VHF, which returns to DHA–DHA through DHA–VHF with two different half-lives.^87^

Petersen *et al.* investigated multiphotochromic systems that connect two DHA/VHF units through either a *para*-phenylene (40) or *meta*-phenylene (41) bridge (Fig. 16).^87^ The linkage configuration of the photoswitch units has a significant influence on the extent to which the two DHA units communicate, and whether each unit can be addressed selectively. Both 40 and 41 are photochromic in MeCN, CH_2_Cl_2_, PhMe and cyclohexane, forming a new absorption band around 460 nm upon irradiation with light, characteristic of VHF formation. These VHFs then undergo thermally induced ring closures back to DHA, though each compound differs in their photoactivities due to the connectivity at the bridging central phenyl ring. The linearly conjugated *para*-phenylene bridged DHA–DHA 40 was reluctant to undergo ring opening and required a more intense lamp with a broader wavelength spectrum and higher radiation energy to achieve full conversion to the higher energy VHA–VHA configuration.

The rates of the stepwise ring openings, or the forward reaction, can be monitored by the increase in the characteristic, red-shifted VHF absorption peak, modelled to an exponential expression giving two time constraints (*τ*_1_ and *τ*_2_), whilst the rate of the thermal back reaction is characterised by the half-life times (*t*_1/2_). Taking the ratio of *τ*_1_ to *τ*_2_ thus gives an approximation of the ratio between quantum yields of the first and second ring openings. These data points for compound 40 could be fitted by one exponential, indicating that the two DHA units are opened non-selectively with similar, very small QYs. ^1^H-NMR spectroscopic studies showed the presence of all three species, namely DHA–DHA, DHA–VHF and VHF–VHF. On the other hand, a cross-conjugated *meta*-arrangement works to separate the two DHA entities, allowing them to undergo stepwise ring openings (Fig. 16), with rates that depend upon the polarity of the solvent; more polar solvents facilitate the sequential switching. These sequential light-induced ring openings of the *meta*-isomer were explained by a significantly reduced photoactivity of the DHA unit when a neighbouring VHF electron acceptor is present, as calculations and the solvent polarity dependence of the switching indicate that the excitation has significant charge-transfer character instead of being a localised DHA excitation. These results highlight the importance of the connectivity in multiphotochromic DHA/VHF systems.

**Fig. 17:**
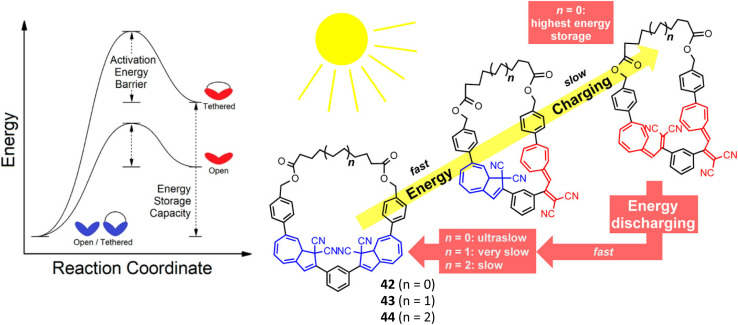
Left: Simplified potential energy surface for acyclic and cyclic, dimeric systems converting between DHA–DHA (blue) and VHF–VHF (red) isomers. Right: Macrocyclic DHA dimers studied by Mikkelsen and Nielsen *et al.* with various ring sizes.^88^ Figures reproduced from ref. 88 with permission from the American Chemical Society.

Another important property when considering the photoswitching capability of the DHA/VHF couple is the pre-equilibrium state between the s-*cis* and s-*trans* conformers of VHF. Whilst the latter is usually more thermodynamically stable, it is only the s-*cis* conformer that has the necessary geometry for ring closure. The idea was proposed that the energy storage density of DHA dimers could be increased by ‘locking’ the VHF into the s-*cis* conformation by means of a small macrocycle with high ring strain for the s-*trans* isomers, a similar strategy as seen above for the azobenzene macrocycles. A potential downside of this is that promoting the s-*cis* conformation has been shown to strongly increase the rate of the thermal reversion, *i.e.*, leading to decreased storage times. Larger macrocycles may work to tune the position of the equilibrium towards the s-*trans* conformer, *via* macrocyclic constraints, which would work to enhance the lifetime of the VHF whilst increasing the Gibbs free energy between the light-harvesting DHA and metastable VHF stables as well as providing a method for controlling the energy release (Fig. 17). Research into DHA dimers for energy storage purposes has been mostly focused on macrocyclic structures for these reasons.

Mikkelsen and Nielsen *et al.* have studied various macrocycles incorporating two DHA units, with alkyl chain linkers of various lengths to study the effect of macrocyclic ring strain on the photoswitching capability and energy storage properties.^88^ All macrocycles, independent of ring size, exhibit stepwise switching upon irradiation at approx. 355 nm, with the first ring opening (DHA–DHA to DHA–VHF) occurring on a much faster timescale than the second. The energy-releasing ring closures also occur in a stepwise fashion, proceeding slower with decreasing ring size due to the increased ring strain. The *meso* and enantiomeric forms of 42 (*n* = 0) were also studied separately,^30^ and though the second ring opening for each occurred on a similar timescale, the first ring opening was slower for the *meso* form (33 s compared to 17 s for the pair of enantiomers) due to some weak fluorescence. Overall, the macrocyclic structure provides an appealing combination of achieving initially fast energy discharge for immediate needs and a slow consistent energy release for long-term uses. The half-life of the second VHF–DHA conversion is increased considerably in the macrocyclic structures (from hours to days at room temperature), with the smaller ring sizes showing the longest half-lives. Furthermore, computational studies have revealed the smallest macrocycles to have the highest energy storage density potentials. Overall, the calculated Gibbs free energy stored per VHF unit is found to be significantly higher for the macrocyclic compounds than their linear counterparts. Various combinations of s-*cis*/s-*cis*, s-*cis*/s-*trans*, and s-*trans*/s-*trans* of VHF–VHF are possible, each having different Gibbs free energies, with the highest energy storage potential reaching 60 kJ mol^−1^, corresponding to a theoretical energy storage density of 73.8 kJ kg^−1^, which is much lower than those reported for NBD dimers and theoretical values for azobenzene macrocycles (Fig. 26). Despite the advancements made in this field,^86^ the energy storage densities of DHA multimers must be increased, as well as improving the solar spectrum match. Incorporating two different photochromic units may offer an alternative solution to combine the beneficial properties of each photoswitch, a strategy that is discussed below.

## Mixed systems

**Fig. 18:**
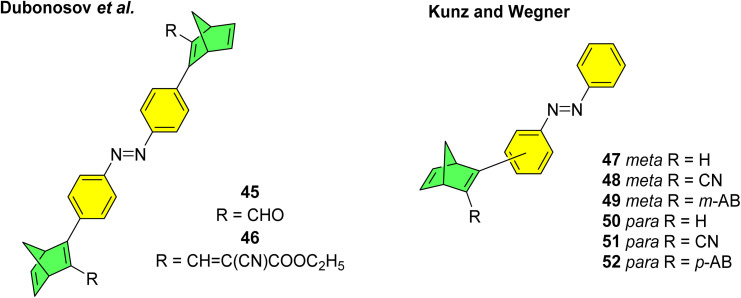
NBD–azobenzene dimers and trimers reported by Dubonosov *et al.*^89^ in 2001 and Kunz and Wegner in 2020.^90^

Various mixed multiphotochromic systems have been investigated that combine two different photoswitch pairs, with the aim of effecting systems in which the complementary strengths of each pair can enhance the photoswitch performance beyond that of the two individual pairs. We have seen that norbornadiene dimers/multimers have perhaps provided the most promise in terms of energy storage densities, though one downside to the NBD/QC couple is that NBD tends to absorb light in the UV region of the spectrum. By combining NBD with azobenzene, the absorption profile may be red shifted whilst also increasing the energy storage density due to the additional energy storage capacity of the azobenzene unit. Dubonosov *et al.* reported the first examples of NBD–azobenzene systems in 2001, combining one azobenzene unit with two NBD units linked at the *para*-position of both phenyl rings of the azobenzene unit to give trimers 45 and 46 (Fig. 18).^89^ As seen above for the azobenzene systems, molecules connected in the *para*-position work to red-shift the absorption and increase the molar extinction coefficient, though they often suffer from low photoisomerization QYs due to the strong electronic communication amongst the azobenzene units. In this study, the authors quote successful switching of both the NBD and azobenzene moieties, though with extremely low QYs of the quadricyclanes of around 0.025 upon irradiation at 436 nm, that could be converted back to the NBD substituents with a molybdenum(vi) oxide heterogeneous catalyst. Impressive absorption long wave boundary values of 500 and 560 nm were reported for 45 and 46 respectively in 2-propanol, though these values have not been specifically defined making it difficult to compare to other absorption onset values. The analysis was limited as no half-life times were reported, and the group only refer to four possible states of the trimers, despite the system potentially offering six states if each NBD unit could be switched selectively also. Nevertheless, this study provided a good initial step towards the study of mixed multi-photochromic systems combining the NBD and azobenzene photoswitch couples.

**Fig. 19:**
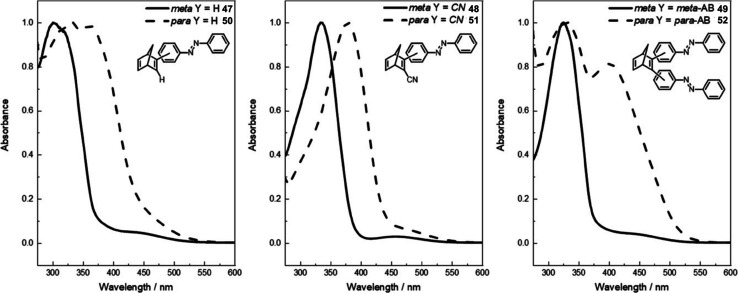
Normalised UV-vis absorption spectra of compounds 47–52 in DMSO (all at 2 × 10^−5^ M except for 47 and 50 which are at 4 × 10^−5^ M).^90^ Reproduced from ref. 90 with permission from John Wiley and Sons.

A further study was published in 2020 by Kunz and Wegner, where four similar NBD–azobenzene dimers and two trimers (49 and 52) were synthesised and studied (Fig. 18).^90^ The effect of the connectivity of the NBD unit(s) to the azobenzene was also investigated by comparing *meta*- and *para*-linked compounds. The UV-vis spectra revealed a red-shift for the *para*-connected compounds 50–52 as expected compared to their *meta*-analogues 47–49 (Fig. 19) due to the elongated π-system for the former. The spectra for 50–52 are also red shifted compared the parent azobenzene and NBD, as well as to an equimolar mixture of the azobenzene and NBD not chemically linked, indicating this electronic communication in the mixed multi-photochromic systems is key in red shifting the absorption. However, upon studying the photoswitching of each compound in various solvents, only *E*-to-*Z* isomerization of the azobenzene unit could be triggered, and no NBD-to-QC isomerization was observed. The equimolar mixture of azobenzene and NBD substituted with a phenyl ring and a cyano group across one double bond was also studied and it was found through ^1^H-NMR spectroscopy that both photoswitches isomerized in acetonitrile-d_3_ under irradiation. These results suggest that energy transfer between both photochromic units due to the conjugation between them inhibits the photoswitching capability of NBD for the multichromophoric systems. This case of intramolecular excitonic coupling has also been observed in other cases of combined photochromic systems^91^ and still poses a problem in the field that must be overcome if these mixed multiphotochromic systems are to be viable for energy storage applications.

**Fig. 20:**
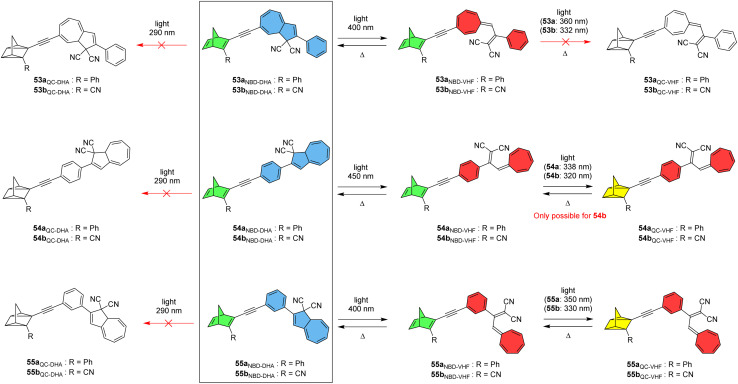
NBD–DHA conjugates reported by Nielsen and co-workers^15^ showing the possible photoisomerization pathways. Note: compounds in colour depict the states that could be accessed.

Nielsen and co-workers created a new class of hybrid MOST chromophore systems by linking together NBD and DHA photoswitches to give six conjugates 53–55a and b (Fig. 20), a rare combination involving both traditional (DHA) and inverse (NBD) chromophores that are both capable of storing energy.^15^ The optical and switching properties were studied in detail by UV-vis absorption spectroscopy in toluene. Linear conjugation between the NBD and DHA units gave the most red-shifted absorption maxima due to a strong interaction between the two units, as discussed previously. Upon irradiation around the longest wavelength absorption onset to target the respective DHA subunits, all conjugates 53–55 were found to easily undergo photochemically induced ring-opening of the DHA scaffold to generate the NBD–VHF form, as confirmed by UV-vis spectroscopy and ^1^H-NMR studies. The ring closures to convert back to the initial NBD–DHA states were successfully achieved thermally for all compounds, with half-lives of 50–70 min for 54b and 55b, and a longer half-life of nearly 600 min for 53b due to the electron-withdrawing alkyne linker attached to the seven-membered ring.^92^ After accessing the NBD–VHF states, the QC–VHF states were targeted by irradiating the samples in toluene at the local maxima (values given in Fig. 20). The QC–VHF states were formed for 54b, 55a and 55b, to give either full conversion or a photostationary state between the QC–VHF and NBD–VHF states, with the half-lives of the QC–VHF compounds strongly dependent upon the solvent, ranging from 11 min in toluene to only 3 min in CH_2_Cl_2_. For 53a, 53b and 54a, no sign of isomerization to the QC–VHF state was observed, originating from an intramolecular effect as the respective uncoupled NBD and DHA could be successfully fully photoisomerized. These findings were rationalized by computational studies by looking at the calculated natural transition orbitals (NTOs). The QC form of each compound could only be accessed by first photoisomerizing the DHA unit to VHF, and then targeting the NBD-to-QC conversion, as irradiation always resulted in conversion of DHA to VHF. It even proved impossible to reach the QC–DHA isomer using a previously published Cu(i)-mediated VHF-to-DHA conversion method.^93^ Nevertheless, Cu(i) was found to change the VHF-to-DHA conversion making it occur on a similar timescale to the QC-to-NBD back reaction, allowing the heat release of each unit to be tuned to the same or different timescales.

A computational investigation into the energy storage densities of compounds 53–55 was undertaken and it revealed that the Gibbs free energy of the NBD–VHF conjugates is 8–10 kJ mol^−1^ more than their NBD–DHA counterparts in toluene, whilst the second isomerization into the QC–VHF forms led to a much larger increase in energy by 96–101 kJ mol^−1^. Surprisingly, this energy storage capacity is higher than that of the unsubstituted NBD/QC photothermal switch, which was found to have a storage density of 88 kJ mol^−1^ at the same level of theory in a dielectric medium of toluene. These findings prove that linking together two photochromic units leads to an overall higher energy storage capacity than the sum of the individual photochromic parts, whilst also enhancing the solar spectrum match. The molecular weight of the photoswitch again must be taken into account, and this leads to theoretical energy storage densities of 0.27, 0.24 and 0.27 MJ kg^−1^ (in toluene) for 54b, 55a and 55b respectively, all of which could experimentally be converted into their QC–VHF forms. It would be beneficial to determine the energy storage densities of such conjugates experimentally in the future to further expand on this study.

**Fig. 21:**
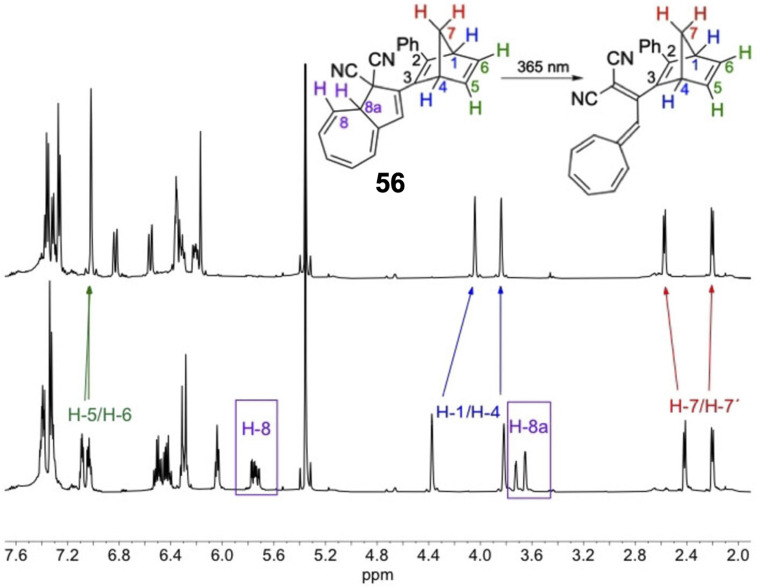
^1^H-NMR spectra at 500 MHz in CD_2_Cl_2_ of NBD–DHA 56 before (bottom) and after irradiation at 365 nm for 10 min producing NBD–VHF 56 (top).^94^ Diagnostic protons from the DHA core disappeared after irradiation whilst the NBD protons only slightly shifted. Reproduced according to Creative Commons CC BY license.

Schøttler *et al.* recently presented a similar NBD–DHA dyad photoswitch where the NBD is directly linked to the DHA unit without the need for the alkyne linker (Fig. 21), thereby reducing the molecular weight and potentially increasing the energy storage density.^94^ Upon irradiation of compound 56 in toluene at 365 nm, a new UV-vis absorption spectrum with a characteristic VHF absorption maximum red-shifted from 365 nm to 466 nm was formed. However, no evidence of QC formation was detected, even after further irradiation of the sample at 254 nm; the ^1^H-NMR spectrum showed the exclusive presence of NBD–VHF. The photoisomerization QY for the DHA-to-VHF conversion was also very low at only 0.05 in toluene, drastically reduced by the proximity of the directly linked NBD moiety. This finding is in line with the lack of photoactivity for some of the *para*-linked NBD–DHA dyads in Fig. 20 due to the strong electronic coupling. A small fluorescence was observed for this compound (56) which likely justifies the loss in photoisomerization quantum yield. Energy storage densities were not calculated for 56, likely due to the QC unit providing the most potential, as seen above. Nevertheless, the half-life of 56 was increased to 5 days at 25 °C, significantly longer than the parent unsubstituted DHA, a property which is desired for solar thermal energy storage applications.

**Fig. 22:**
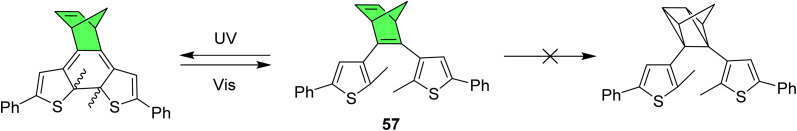
NBD-bridged diarylethene photoswitch shows ring-closure of the DAE but no isomerization of NBD to QC upon irradiation.^95^

Another interesting multiphotochromic system where in which NBD is used to bridge diarylethene has been reported to show fluorescence that can be turned on or off as the molecule photoswitches (Fig. 22).^95^ Diarylethenes (DAE), as shown in Scheme 1, are positive photochromes that form a ring-closed structure upon irradiation with light. For these structures, NBD was used as a bridge to investigate the effects of having a positive and negative photochromic system in one structure in such close proximity. In this case, for all compounds studied, including compound 57 as a representative example, only the DAE could be switched upon irradiation and the NBD could not be converted to QC. Nevertheless, the NBD bridge proved invaluable as the alkene functionality could be utilized to fluorescently tag the photochrome with a fluorescein-modified tetrazine, thereby generating a turn-off mode fluorescent photoswitch. Cyclization and cycloreversion of this DAE-fluorophore conjugate was induced by irradiation with UV-light and visible light, respectively, with fluorescence quenching observed for the closed DAE form.

**Fig. 23:**
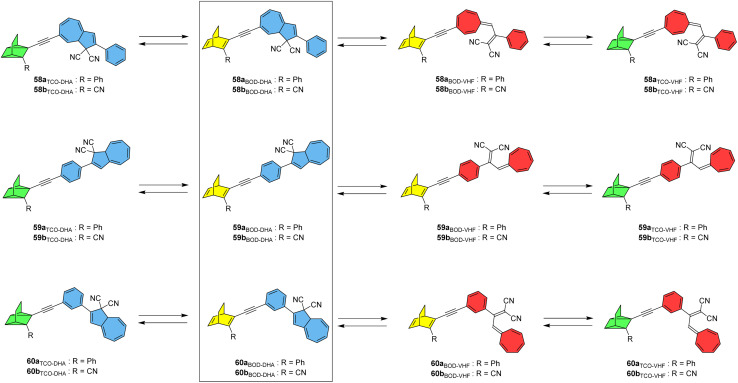
BOD–DHA conjugates theoretically studied for energy storage applications.^98^

A new photoswitch that is similar to NBD is emerging; bicyclooctadiene (BOD) differs to NBD in that it has an extra carbon atom on the bridgehead (Fig. 23).^96,97^ Upon irradiation, BOD can be converted into the corresponding higher energy photoisomer tetracyclooctane (TCO). The photophysical properties of BODs have been less explored than other systems, as TCO is rapidly converted to BOD through thermal activation. A degradation pathway into an aromatic by-product *via* a retro-Diels–Alder reaction also exists and is irreversible due to the loss of gaseous ethene in the process. Nevertheless, some BODs have been isolated and reported in the literature, and the potential storage energy for BOD/TCO is even higher than that of NBD/QC at 1.77 MJ kg^−1^.^96^ A theoretical study by Elholm *et al.* considers a set of photoswitch conjugates that combine the BOD/TCO and DHA/VHF photoswitch pairs, with structures that are identical to the NBD–DHA conjugates presented by some of the same authors in Fig. 20 but with BOD in place of NBD.^98^ Despite the potential storage energy of BOD being higher than NBD, its absorption spectrum is blue shifted, resulting in smaller overlap with the solar spectrum. The well-investigated DHA/VHF system has a much better overlap with the solar spectrum and hence was investigated in combination with BOD/TCO.

The targets of this computational study and their potential isomerization states are given in Fig. 23. Cyano-substituted b-systems have significantly larger storage energy densities compared to the corresponding phenyl-substituted a-systems due to the lower molecular mass of the former. Theoretical analysis of storage energy densities was carried out as per the previous study and it was found that the initial DHA-to-VHF conversion leads to a significant increase in Gibbs free energy of 27 kJ mol^−1^ for system 59b, whilst the BOD to TCO conversion results in a much larger energy increase of 166 kJ mol^−1^. The total predicted storage density of 59b is 0.48 MJ kg^−1^, lower than the unsubstituted BOD/TCO system (1.77 MJ kg)^96^ but still a considerable improvement over the unsubstituted DHA/VHF system (0.11 MJ kg^−1^). These BOD–DHA conjugates overall exhibit a 50% higher storage density than the previously investigated NBD–DHA conjugates.^15^ The highest storage density is predicted for 60b, at 0.483 MJ kg^−1^ in toluene. Whilst DHA and BOD(CN) dissolved individually in toluene would lead to a slightly higher storage density of 0.499 MJ kg^−1^, BOD(CN) does not absorb within the solar spectrum. However, the absorption of the BOD parent structure is red shifted to a longer wavelength when incorporated into a dyad with DHA, resulting in a larger spectral overlap with the solar spectrum, making it more applicable for solar thermal energy storage and conversion. Natural transition orbitals (NTOs) were again considered computationally to aid in estimating the potential conversion from BOD to TCO within the dyad at a given excitation wavelength. The analysis of excitations and NTOs suggests that system 59b has the potential for full conversion through exposure to light. The conversion of DHA to VHF results in a red-shift and increased intensity of the absorption peak corresponding to the BOD unit, which in turn amplifies the overlap of this peak with the solar spectrum and increases the probability of BOD-to-TCO conversion. This suggests that first the DHA unit must be converted before BOD can be converted, mirroring what was observed experimentally for the similar NBD–DHA conjugates. Further investigation into these systems through excited-state molecular dynamics simulations, and/or experimental studies if they could be synthesised, would be beneficial.

**Fig. 24:**
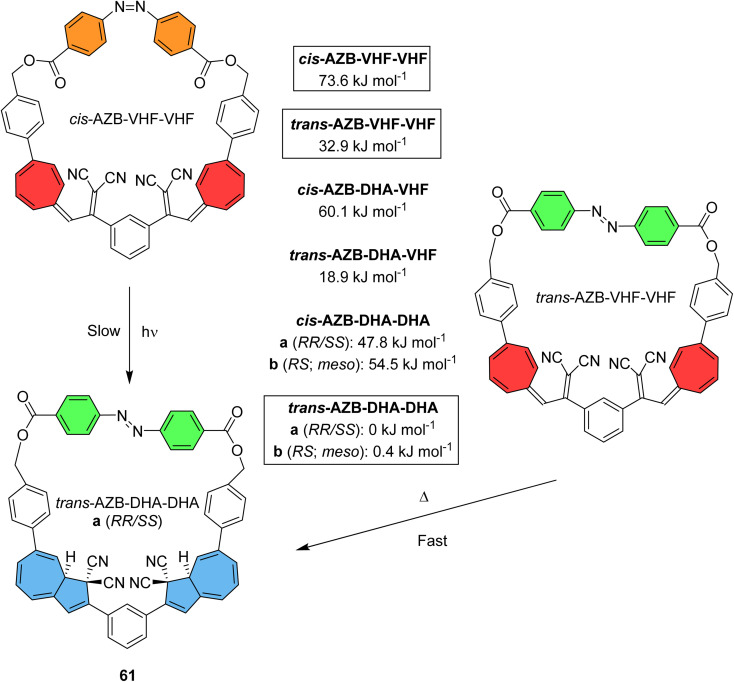
Accessible states of macrocycle 61, along with calculated Gibbs free energies of formation (CAM-B3LYP/6-311G(d,p)) of isomeric macrocycles. Note that the *trans*/*cis*-AZB–DHA–DHA states can each exist as pairs of diastereoisomers (one meso and a pair of enantiomers), and the *trans*/*cis*-AZB–DHA–VHF states can exist as pairs of enantiomers.^21^

Macrocyclic structures featuring either azobenzene or DHA have been discussed, where macrocyclic ring strain can increase the potential energy storage density whilst providing additional control over the available states. By cyclising two DHA units together with one azobenzene, Vlasceanu *et al.* synthesised and studied macrocycle 61 for which at least six distinct isomers may occur (Fig. 24).^21^ Calculations show that the *trans*-AZB–DHA–DHA isomer is the most stable conformation, whilst *cis*-AZB–VHF–VHF is the least stable at 73.6 kJ mol^−1^. This value can be converted to give a theoretical energy storage density of 83.5 kJ kg^−1^ for 61, relatively low due to the high molecular weight of the macrocycle. The group show that three out of the six possible states can be accessed experimentally through light or heat.

The ground state conformer of 61, *trans*-AZB–DHA–DHA, was irradiated with light at 365 nm to give *cis*-AZB–VHF–VHF, reaching a photostationary state of 86%. As *cis*-AZB is photochromic and VHF is thermochromic, the energetically more stable *trans*-AZB–VHF–VHF state could also be accessed by irradiation at 410 nm where the *cis*-AZB absorbs. Thermal isomerization of VHF–VHF to DHA–DHA could be initiated from two starting points, either *trans*-AZB–VHF–VHF or *cis*-AZB–VHF–VHF. Both processes were monitored at various temperatures in MeCN and in each case the photoisomerization was observed to occur stepwise *via* an intermediate DHA–VHF state. Relaxation of *cis*-AZB–VHF–VHF to *trans*-AZB–DHA–DHA was observed to initially proceed *via* a fast decay (*t*_1/2_ = 36 min at 30 °C) followed by a much slower process (*t*_1/2_ = 1900 min at 30 °C). The first event is assigned to first the conversion of one VHF unit to DHA. The relaxation of *trans*-AZB–VHF–VHF to *trans*-AZB–DHA–DHA also proceeded *via* a fast and a slow thermal decay, accounting for two VHF-to-DHA isomerizations with half-lives of 31 and 620 min at 30 °C. By comparing the two possible pathways from the highest energy isomer to its ground state conformation, the first VHF-to-DHA conversion was shown to be unaffected by the state of the azobenzene. The second ring closure, however, is largely dependent on the neighbouring azobenzene state, as it is three times faster for *trans*-AZB compared to *cis*-AZB. Photoisomerizing the *cis*-AZB to *trans*-AZB first, speeds up the thermal regeneration of *trans*-AZB–DHA–DHA. This macrocylic multichromophoric photoswitch presents a convenient way of controlling accessibility of specific states.

**Fig. 25:**
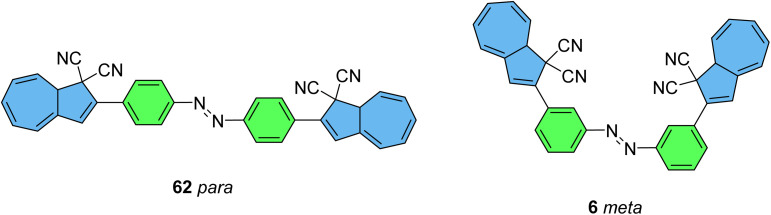
Triad DHA–AZB–DHA systems with *para* (62) and *meta* (63) connectivities studied by Mengots *et al.*^99^

The conjugation between the DHA and AZB photoswitches in 61 is broken by sp^3^-hybridised carbon atoms, keeping the units as separate chromophores. Some of the same authors therefore explored trimeric photoswitches 62 and 63, where two DHA units in this case are conjugated to an azobenzene unit with either *para*- or *meta*-connectivity (Fig. 25), to investigate the effect of having these two photoswitches coupled in close proximity and with different connectivities.^99^ The UV-vis absorption spectrum of compound 62 in CH_2_Cl_2_ peaks at 426 nm, red-shifted in comparison to DHA-Ph at 357 nm (Scheme 1, R = Ph) and to unsubstituted azobenzene at 320 nm, as expected due to the extended conjugation of both chromophores. The electronically coupled *para*-compound 62 undergoes reversible *trans*-to-*cis* isomerization of the azobenzene unit upon irradiation at 415 nm, as well as possibly some irreversible sigmatropic transformation of 1,8a-DHA into 1,3a-DHA, though ultrafast experiments did not allow the complete identification of this photoproduct. No DHA-to-VHA conversion is observed due to the lack of a selective DHA excitation, as the frontier molecular orbitals are distributed all over the *para*-conjugated part of the molecule. The absorption of compound 63 is less red shifted (*λ*_max_ = 358 nm) due to the broken conjugation between the chromophores. Irradiation at 365 nm in this case triggers the DHA-to-VHF conversions, as well as some *trans*-to-*cis* isomerization of the azobenzene unit forming a photostationary state, due to more localised orbitals involved in the transition at one DHA unit whilst also containing part of the AZB unit. Computational studies reveal that the units are not entirely decoupled in 63. Both the steady-state study and detailed transient absorption spectroscopy study revealed that the photoisomerization of the DHA and AZB moieties occur in a stepwise manner, with an ultrafast DHA-to-VHF conversion followed by a slower *trans*-to-*cis* AZB isomerization. In addition, the thermal back reaction occurred at the same rate for all VHF units, independent of the isomeric state of the neighbouring units in the triad. This shows that connectivity is crucial for the design of multiphotochromic systems, especially when they are in direct conjugation with one another. Once again, it also highlights the difficulty in designing multiphotochromic systems where all states can be accessed.

## Summary and discussion

This review highlights the current status of multichromophoric systems incorporating photoswitches such as NBD, azobenzene, DHA, or a combination of chromophores, for energy storage applications. Multichromophoric photoswitches can offer several advantages over monosubstituted analogues, such as red-shifted absorption profiles due to the extended conjugation. This is highlighted in Table 2 where select multichromophoric systems are compared to analogous single chromophoric switches. For each NBD multichromophoric system, a beneficial red shift of approx. 40 nm is observed in the absorption onset compared to analogous single chromophores. A similar trend is observed for the DHA *para*-conjugated systems, whereas for azobenzenes the red shift in absorption is only small (around 10 nm) upon the incorporation of more chromophore units.

**Table 2:** Properties of select multichromophoric photoswitches compared to analogous single chromophores

Photoswitch	ref.	Number of chromophore units	*λ*_onset_/nm	QY	*t*_1/2_	Δ*H*/kJ mol^−1^	Energy density/kJ kg^−1^
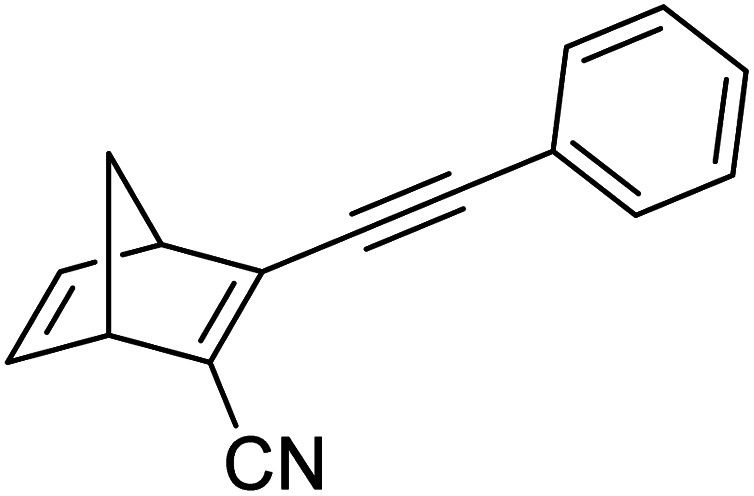	27	1	374^b^	0.39	22 h	102	469
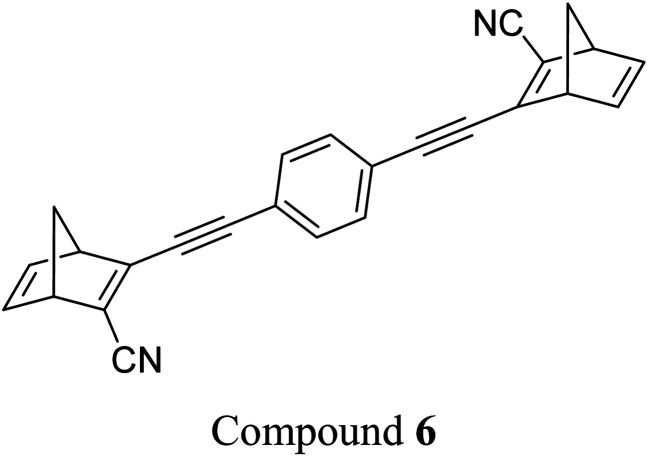	13	2	411^c^	0.94	4.33 h (QC–QC)	183.3	514.2
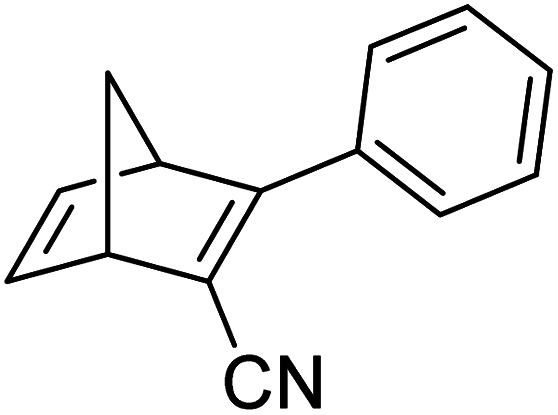	27	1	358^b^	0.58	1320 h	122	629
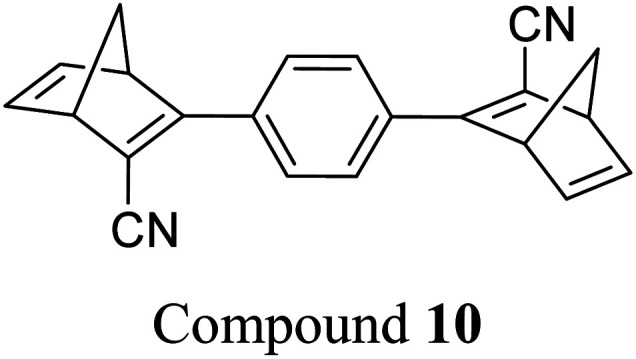	13	2	400^c^	0.73	10.6 days (QC–QC)	237.9 (calculated)	771.4 (calculated)
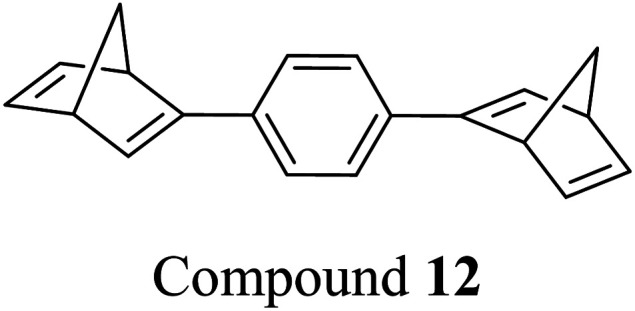	31	2	370^c^	0.56	2 days	175	678
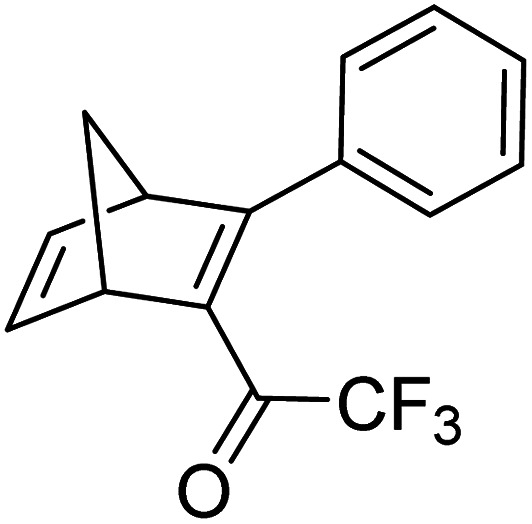	101	1	426^b^	0.53	72 h	152	575
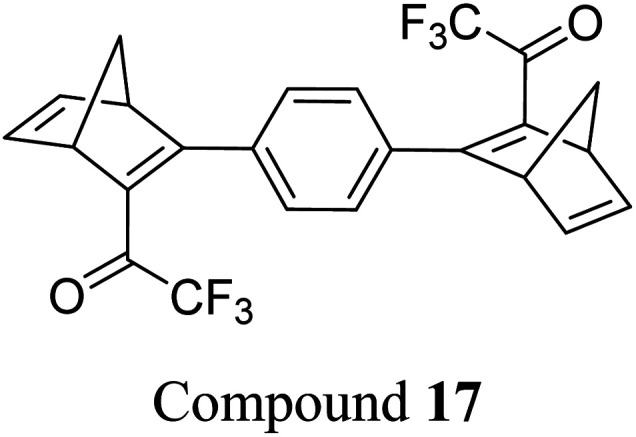	25	2	466^b^	0.77	12 h (QC–QC)	146.8	326
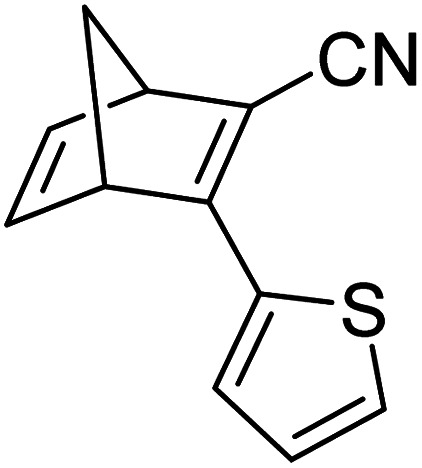	100	1	390^b^	0.61	0.22 days	114	571
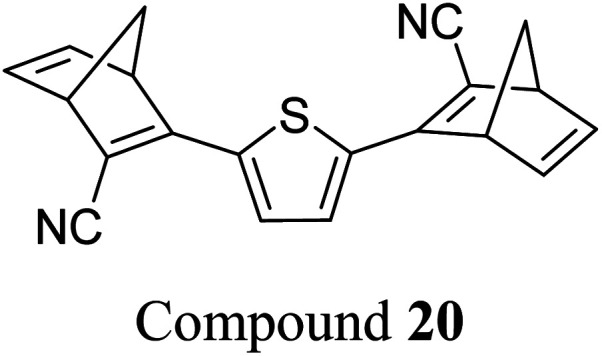	32	2	420^a^	0.44	10.7 min	—	—
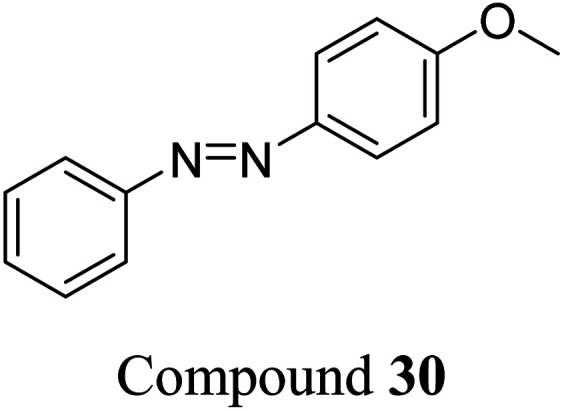	69	1	347 (n–π^a^ band) d	0.23	37.7 min	54.1	255
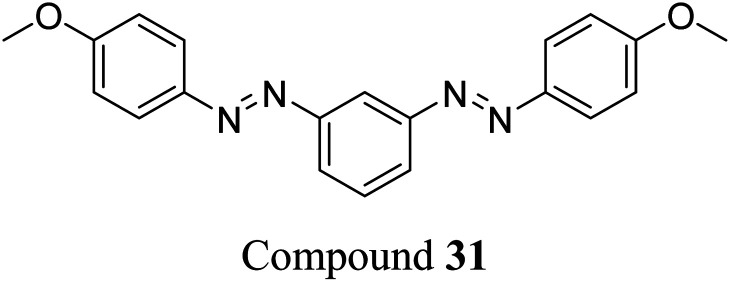	69	2	357^a^	0.11	28.1 h	94.2	272
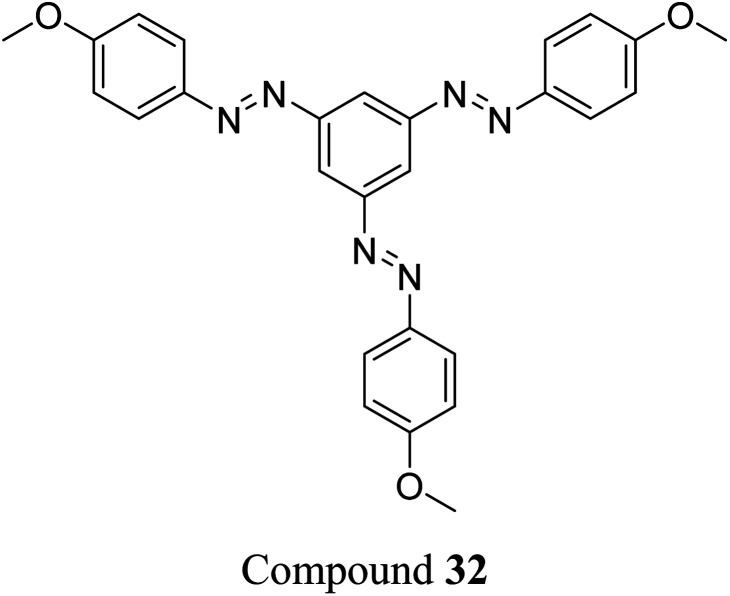	69	3	362^a^	0.09	20.5 h	116	242
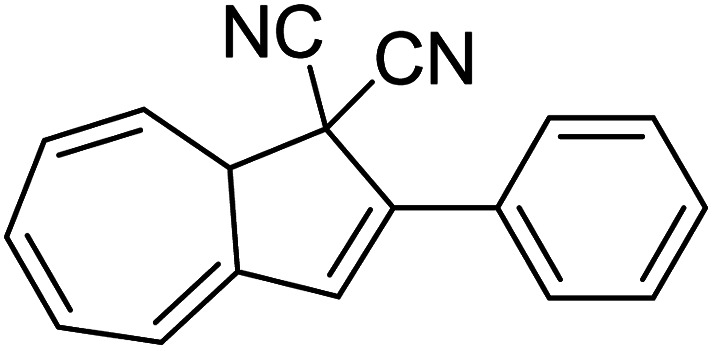	87	1	355 (*λ*_max_)^e^	—	216 min	—	—
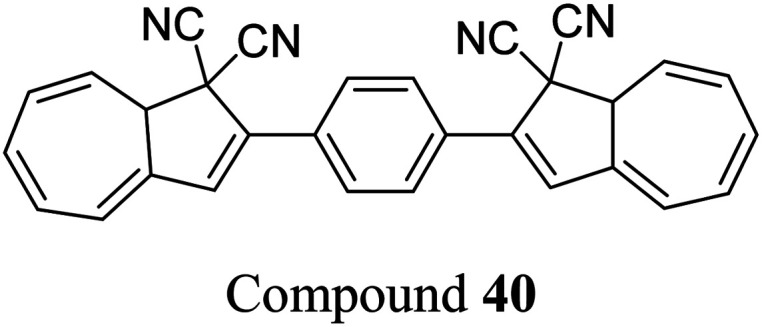	87	2	408 (*λ*_max_)^e^	—	—	—	—
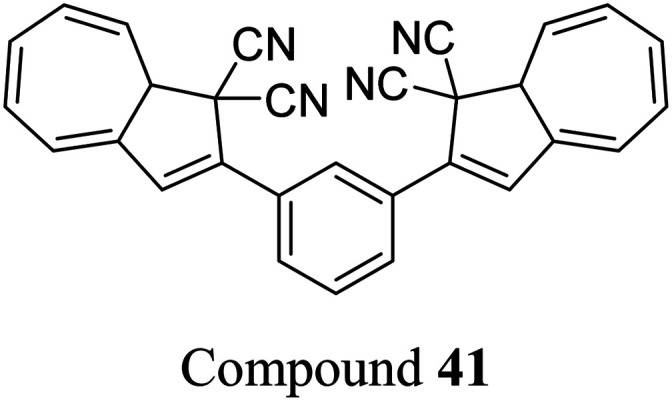	87	2	365 (*λ*_max_)^e^	—	160 min	—	—

a Values extracted manually from the reported spectra. Solvents used for measurements.

b Toluene.

c Cyclohexane.

d THF.

e MeCN.

Although these multichromophoric systems can offer several advantages, designing the ‘ideal’ MOST system is intrinsically challenging as it is a multidimensional problem, and optimising some properties often comes at the expense of others. For example, a red shift in the absorption profile has been known to lead to decreased storage times. This can be explained by considering the coupling between the S_0_ and S_1_ energy landscapes. Whilst the HOMO and LUMO have π–π and π* + π* on the NBD side of the reaction profile, they have σ–σ and σ* + σ* character in the case of QC, with the intramolecular cycloaddition reaction to convert NBD to QC involving an avoided crossing of HOMO and LUMO levels. Reducing the S_0_–S_1_ spacing for NBD therefore usually implies a lowering of the barrier for thermal back-conversion.^100^ Multichromophoric systems can be a way of improving the storage times compared to single chromophoric switches due to the multistate nature, as exemplified by compounds 10 and 11 (Table 2),^13^ though this is not always the case, as observed with other entries in Table 2. For example, the half-life values for 30, 31 and 32 decreased with increasing number of azochromophores, with values of 37.7, 28.1 and 20.5 h respectively. Other strategies to increase storage times could be used in combination, for example adding *ortho*-substituents to the phenyl rings coupled to NBD, to design multichromophoric switches with potentially better properties.^100^

Double systems can also lead to increased energy storage densities, in particular for the NBD systems due to the blue shift in absorption that occurs after the first isomerization step. Record high energy storage densities have been observed for NBD oligomers,^13^ in particular compound 6 has a heat release value of 183 kJ mol^−1^ compared to 102 kJ mol^−1^ for the analogous mono-substituted NBD (Table 2), with corresponding energy densities of 514 and 469 kJ kg^−1^ respectively.^27^ This can be due to the fact that some of the molecular weight is ‘shared’ across two or more photoswitch units in the case of the multichromophoric systems, leading to higher energy densities. In the case of azobenzene systems, increasing the number of photochromic units does not necessarily improve the energy storage properties; the dimer 31 has a higher storage density than the mono 30, but an additional photochromic unit led to a decrease in the energy storage for 32 due to increased steric hindrance and a low degree of isomerization. Moreover, as discussed previously, the calculated theoretical solar capture efficiency is less than 1% for azobenzene systems.^49^

**Fig. 26:**
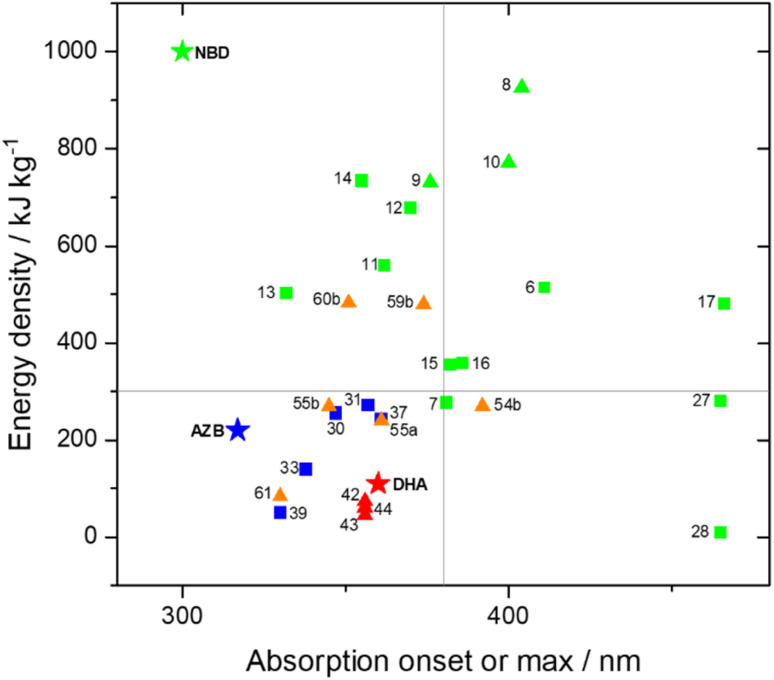
Energy densities of multichromophoric photoswitches presented in this review and their absorption onset (for NBD compounds) or maxima (for AZB, DHA and mixed compounds) as defined in the original literature data. Key: green – norbornadiene (NBD) containing compounds;^13,22,25,31,43^ blue – azobenzene (AZB);^46,63,67,69,84^ red – dihydroazulene (DHA);^85,88^ orange – mixed systems.^15,21,98^ Stars represent the parent compounds; squares represent experimental results and triangles theoretical values. The horizontal line represents the target energy density of 300 kJ kg^−1^ whilst the vertical line represents the start of the visible region of the solar spectrum.

Fig. 26 provides a comparison between all multichromophoric photoswitches presented in this review that have a reported experimental or theoretical energy density, with the unsubstituted parent NBD, DHA and AZB compounds included for comparison. Of course, there are many parameters that must be considered when comparing these molecules for energy storage purposes, most of which can be incorporated together to give an overall efficiency of such systems.^12,102^ For example, the calculated solar capture efficiency of 17 is 3.8%,^25^ compared to an experimentally achieved value of 1.1% from a single NBD system.^103^ This shows that energy density cannot be used alone as a measure of the efficiency of such systems, as 17 has a lower energy storage density than the respective single chromophore (Table 2), but its red-shifted absorption and higher photoisomerization QY contributes to the large calculated solar capture efficiency. There are also limits on how efficient such systems can become, due to factors such as the solar spectrum match and the barrier for the back reaction (Δ*G*^‡^).^102^ Nevertheless, with the help of multichromophoric switches, MOST systems are gradually being developed towards the outlined theoretical goal of 12% efficiency for a photoswitch with a half-life of 24 days.^102^

However, energy density values are more commonly reported, and so these values are presented here (Fig. 26) as a measure of the potential of each candidate for energy storage applications. These energy density values are plotted against the reported absorption onset values of the ground state molecules, as defined in the original data sets, for the NBD oligomers. As alluded to previously, these onset values can vary between research groups who define the onset slightly differently, whether that is where *ε* is roughly 0, or where the absorption is slightly more pronounced. Nevertheless, these values provide a guideline to the absorption limit of each compound. For the azobenzene and DHA compounds, the absorption maximum is plotted, as this value is widely reported and accepted for positive photochromic molecules and is more meaningful as the absorption of the higher energy isomers would overlap with the ‘onset’ of absorption for the ground state. Despite these limitations and differences, this graph should help provide a comparison of all compounds reviewed and schematically highlight what energy density and absorption values have been achieved thus far.

Perhaps the multichromophoric systems incorporating NBD have shown the most potential in terms of high energy storage densities compared to azobenzene and DHA systems (Fig. 26), pushing towards the theoretical limit outlined by Börjesson and co-workers.^102^ Other advantages of the NBD system include the ability to catalytically trigger the back reaction, whereas for azobenzenes the back reaction is triggered by light. This means full conversion is difficult to achieve for azobenzenes, leading to photostationary states. Strategies to overcome this have been discussed, for example using gold nanoparticles (see Fig. 12) to stimulate the discharging procedure by light and the thermal energy generated by the photothermal effect of the nanoparticles. Introducing ring strain to give macrocyclic structures has also been the most effective strategy to improve the energy storage properties of multichromophoric azobenzene systems. With DHA, full conversion between the two states (DHA and VHF) is possible as the system is photoactive in the forward direction and thermally active in the other. The calculated energy density of the parent unsubstituted DHA/VHF system is less than that of NBD (0.11 MJ kg^−1^ compared to 1 MJ kg^−1^), though combining both DHA and NBD into one system increased the theoretical energy storage compared to DHA alone (Fig. 26). This particular study highlights that linking together two photochromic units can lead to overall higher energy storage capacities than the sum of the individual photochromic parts, whilst also enhancing the solar spectrum match, though the molecules in that study have not yet been synthesised and studied experimentally. Further theoretical studies where in which the NBD units are replaced by bicyclooctadiene switches (BOD) conjugated to DHA have also shown promising theoretical energy storage densities, up to 50% greater than their NBD counterparts.^98^

Although the multichromophoric switches presented here have shown great potential for energy storage, they have yet to been investigated in MOST devices (though 17 has been studied in film for window coating applications),^25^ which is something that would be exciting to explore in the future. In order to achieve this, one must consider the scalable synthesis of such systems. For norbornadiene compounds, the Diels–Alder reaction is a preferable synthetic route as this can be scaled using flow chemistry techniques,^28^ as previously discussed. Recently, a flow route to the acetylene precursors required for the Diels–Alder step has also been recently developed for single chromophore NBDs.^29^ Intuitively, more complex multichromophoric designs can potentially cause problems with scalability of the synthesis and this is something that must be considered. Stability must also be considered, which is usually investigated by performing cyclability studies with continuous switching between the parent and photoisomer. To be stable enough for applications, it is desirable to perform several cycles with minimal degradation of the photoswitch. A future goal could be to have systems that can withstand up to 10 000 cycles, corresponding to operation in a MOST device every day for 30 years. Current research efforts in the MOST field are focused on improving molecular design,^104^ with some new motifs^105^ that could be applied to multichromophoric switches in the future. Other strategies involve using triplet–triplet-annihilation up-conversion (TTA-UC)^106,107^ to overcome the limitation of absorption in the UV region of the spectrum.^108^ Utilisizing photosensitizers can also be an effective strategy as discussed for the TADF compounds 27–29,^43^ altogether highlighting a breadth of opportunities that can be used to develop systems with performance beyond todays state-of-the-art.

## Conclusions

In this review, we have summarized multichromophoric photoswitches as candidates for energy storage applications. These multichromophoric photoswitches offer several advantages over their monosubstituted counterparts, namely red-shifted absorption profiles due to the extended conjugation, as well as increased potential energy storage densities per molecule due to some of the molecular weight being ‘shared’ across each unit. The highest reported energy densities so far are seen amongst NBD oligomers, particularly a theoretical value of 927 kJ kg^−1^ for a dimeric system,^13^ and an experimental value of 734 kJ kg^−1^ for a molecule featuring three NBD units,^31^ although there is still room for improvement as this trimer system absorbs only towards 386 nm. Incorporating donor–acceptor character, such as by the use of cyano groups into the structure is a good strategy to red shift the absorption profile so that it better overlaps with the solar spectrum, but this comes at a cost of increasing the molecular weight of the system which in turn decreases the energy storage density per kilogram of material. Azobenzene oligomers have been more extensively studied, as well as molecules featuring the dihydroazulene (DHA) switch, particularly incorporated into macrocyclic structures to control the geometry and hence potential energy storage densities and lifetimes of the systems. The properties of DHA and azobenzene oligomers can be improved by incorporating these positive photochromes with the negative photochromic NBD switch to give mixed multichromophoric systems that can combine the advantages of each individual photoswitch. However, these systems often pose additional problems for example loss of photochromism in one part of the switch. Overall, we highlight that there are multiple factors at play that govern the efficiency and switching properties of these multichromophoric systems for energy storage purposes, in particular the connectivity of each unit. There are still improvements to be made in the search to find the ‘ideal’ system. Nevertheless, the efforts produced so far in this field have established multichromophoric systems as promising candidates for energy storage applications amongst others.

## Conflicts of interest

There are no conflicts to declare.

## Supplementary Material
